# A network meta-analysis of the performance of acupoint stimulation therapy in improving fatigue, neurological function, and activities of daily living in patients with multiple sclerosis

**DOI:** 10.3389/fneur.2026.1796876

**Published:** 2026-05-26

**Authors:** Ruiou Chen, Hongyu Li, Yuwei Ben, Boying Zhang, Qiang Tang

**Affiliations:** 1 Heilongjiang University of Chinese Medicine, Harbin, Heilongjiang, China; 2 The Second Affiliated Hospital, Heilongjiang University of Chinese Medicine, Harbin, Heilongjiang, China

**Keywords:** activities of daily living, acupoint stimulation therapy, fatigue, multiple sclerosis, network meta-analysis, neurological function

## Abstract

**Introduction:**

Multiple sclerosis (MS) is a chronic autoimmune disease with a high disability rate, primarily characterized by inflammatory demyelination of the central nervous system. As a complementary and alternative therapy, acupoint stimulation exhibits potential in alleviating the symptoms of MS. However, the relative efficacy of different therapies remains unclear. This study sought to systematically compare the effects of various acupoint stimulation therapies on neurological function, fatigue, and activities of daily living in MS patients through a network meta-analysis (NMA).

**Methods:**

Eight databases were systematically searched for relevant publications from their inception to August 3, 2025. Randomized controlled trials (RCTs) comparing different acupoint stimulation therapies (e.g., acupuncture, electroacupuncture, moxibustion, acupressure, acupoint injection) for treating MS were incorporated. A Bayesian NMA was implemented to comprehensively compare the impacts of various interventions on Expanded Disability Status Scale (EDSS) scores, Fatigue Severity Scale (FSS) scores, the Barthel index (BI), and response rates. The surface under the cumulative ranking curve (SUCRA) values were utilized to evaluate the relative ranking of each regimen.

**Results:**

Finally, 23 RCTs involving 1,384 patients and 8 acupoint stimulation interventions were incorporated. The results suggested that electroacupuncture combined with acupoint injection demonstrated the greatest effectiveness in improving neurological function (EDSS) (mean difference [MD] = −2.9, 95% CrI: −3.4 to −2.3). Acupressure therapy performed the best in relieving fatigue (FSS) (standardized mean difference [SMD] = −1.3, 95% CrI: −1.6 to −1.0). Electroacupuncture combined with acupoint injection demonstrated the best efficacy in improving the activities of daily living (BI) (MD = 18, 95% CrI: 8.8 to 28).

**Conclusion:**

The application of acupoint stimulation therapy in combination with standard pharmacotherapy has demonstrated potential value in relieving fatigue, improving neurological function, and enhancing quality of life in patients with MS. However, due to methodological limitations of the included studies and clinical heterogeneity among interventions, the findings of this study should be considered exploratory evidence and are insufficient to support definitive clinical recommendations. This study aimed to provide a basis for hypotheses and a reference for intervention prioritization for future research. Therefore, the conclusions should be interpreted with caution.

**Systematic review registration:**

https://www.crd.york.ac.uk/PROSPERO/display_record.php?RecordID=51124476, PROSPERO CRD420251124476.

## 1 Introduction

Multiple sclerosis (MS) is an autoimmune disease characterized by inflammatory demyelination of the central nervous system (CNS) (1, 2). Major clinical manifestations of MS include visual impairment, limb weakness, sensory abnormalities, ataxia, autonomic dysfunction, and cognitive impairment (3). MS is a lifelong condition with dissemination in space and time and worsens gradually. Its associated complications and functional impairments often aggravate the physical and psychological burden on patients, substantially elevate the risk of disability, and seriously impact patients’ quality of life. Therefore, reducing the degree of disability, preventing complications, and improving patients’ quality of life are key objectives in MS management (4).

The long-term management of MS primarily involves a comprehensive standard strategy that includes disease-modifying treatment (DMT), treatment for acute recurrence, and symptomatic and supportive care (5–9). DMT is central to the long-term management of MS. Initiating DMT as early as possible in diagnosed patients can control inflammation before irreversible neurological damage occurs and reduce long-term disability rates. The standard treatment for acute recurrence of MS is high-dose methylprednisolone pulse therapy, which rapidly reduces inflammation, alleviates symptoms, and shortens the duration of the disease. Additionally, symptomatic and supportive care is crucial for improving patients’ quality of life. Symptom management in MS involves targeted pharmacological interventions for core symptoms such as spasticity, neuropathic pain, and fatigue, combined with rehabilitation management for bladder or rectal dysfunction.

Meanwhile, given the potential synergistic effects and favorable safety profile of adjunctive therapies, an increasing number of patients tend to use them as an adjunct or integrative component of existing standard treatments (10). Acupoint stimulation therapy, a core treatment method in traditional Chinese medicine (TCM), achieves therapeutic effects by applying physical stimulation to specific acupoints on the body surface to regulate qi and blood and balance yin and yang. Acupoint stimulation therapy encompasses both invasive techniques, such as acupuncture and electroacupuncture, and non-invasive techniques, such as moxibustion and acupressure. It is safe, user-friendly, and cost-effective. In recent years, acupoint stimulation therapy has exhibited unique potential in symptom management and supportive treatment for MS. A randomized controlled trial (RCT) has found that electroacupuncture, used as an adjunctive to immunomodulatory therapy, can alleviate pain and depressive symptoms in patients with relapsing remitting MS (RRMS), thereby improving their quality of life (11). An RCT from Turkey has also revealed that acupressure therapy, administered in conjunction with conventional treatment, can reduce MS-related fatigue (12). Furthermore, basic research has explored the underlying neurobiological mechanisms and found that acupuncture can reduce the release of pro-inflammatory cytokines such as interleukin-6 and tumor necrosis factor alpha. Electroacupuncture can mitigate the inflammatory injury of the myelin by modulating the phenotype of macrophages (13). It can also promote the phagocytosis of myelin fragments by microglia, thereby accelerating the regeneration and repair of the myelin after demyelination (14).

Incorporating diverse acupoint stimulation therapies into the long-term management strategies of MS brings numerous benefits to patients. However, comparative evidence evaluating the relative efficacy of various acupoint stimulation methods remains insufficient to support clinical decision-making. Therefore, this study employed a network meta-analysis (NMA) approach to comprehensively evaluate and compare the relative efficacy of different acupoint stimulation therapies across three outcome measures for MS: neurological function (Expanded Disability Status Scale [EDSS]), fatigue severity (Fatigue Severity Scale [FSS]), and activities of daily living (Barthel index [BI]). It was expected to provide guidance for clinical practice and future research.

## 2 Methods

This study was implemented in adherence to the Preferred Reporting Items for Systematic Reviews and Meta-Analyses Extension Statement for Reporting of Systematic Reviews Incorporating Network Meta-Analyses of Health Care Interventions (PRISMA-NMA) (15). The study was pre-registered in the International Prospective Register of Systematic Reviews (PROSPERO) (CRD420251124476).

### 2.1 Data sources

Cochrane Library, Embase, PubMed, Web of Science, China National Knowledge Infrastructure (CNKI), Wanfang Data Knowledge Service Platform (Wanfang Data), Weipu Chinese Scientific and Technical Journal Database (VIP), and China Biomedical Literature Database (SinoMed) were systematically searched for publications from the establishment of each database to August 3, 2025. The search strategy was designed by using combinations of subject headings and free-text terms, including MS, acupuncture, acupuncture therapy, cupping therapy, electroacupuncture, ear acupuncture, acupoint catgut embedding therapy, acupressure, fire acupuncture, and heat acupuncture. The search strategies for the databases are detailed in Supplementary Table 1.

### 2.2 Study selection

Two reviewers (C.R. and B.Y.) independently screened the literature, and a third reviewer (Z.B.) consolidated the results. Inclusion criteria were developed based on the Population, Intervention, Comparison, Outcomes, and Study design (PICOS) framework as follows: (i) Population: Patients diagnosed with MS according to the McDonald criteria (16–18), regardless of race, sex, or disease duration, aged 18 years or older; (ii) Intervention: Acupoint stimulation therapies including acupuncture, electroacupuncture, acupressure, moxibustion, and warm needling, administered in addition to standard treatment; (iii) Comparison: Standard treatment (The specific standard treatments used in included studies are presented in Supplementary Table 2) or sham stimulation; (iv) Outcome: response rate (The specific definitions of response rate in included studies are presented in Supplementary Table 3), EDSS score, FSS score, BI for Activities of Daily Living (ADL); and (v) Study design: RCT.

Exclusion criteria included: (i) Various reviews, case reports, summaries of experience, animal studies, observational studies, and commentaries; (ii) Duplicate publications or studies; and (iii) Studies with incomplete key data.

### 2.3 Risk of bias assessment

The risk of bias was evaluated by employing the National Institutes of Health (NIH)/National Heart, Lung, and Blood Institute (NHLBI) tools for Quality Assessment of Controlled Intervention Studies.^1^ The evaluation covered randomization methods, allocation concealment, blinding implementation, baseline comparability, loss to follow-up, compliance, other interventions and controls, outcome measurement, sample size calculation, pre-specified outcomes, and intention-to-treat analysis. Based on a comprehensive assessment of potential risks of bias for each item, the incorporated studies were ultimately rated as high-, moderate-, or low-quality. Two researchers (C.R. and B.Y.) independently evaluated the quality of each incorporated study. If any disagreement on the inclusion of a study occurred, a third researcher (Z.B.) was consulted, and a final consensus was reached.

### 2.4 Data extraction

Data were extracted independently by two researchers (C.R. and B.Y.). In case of any disagreement, the data involved were extracted by a third researcher (Z.B.) until a consensus was reached. A pre-designed basic information extraction form was utilized to extract data, encompassing: (i) Study details: first author, publication year, region/country, study type; (ii) Baseline characteristics of study populations: sample size, sex ratio, age, MS subtype; (iii) Interventions: detailed documentation of specific acupoint stimulation methods, acupoint selection, intervention duration, frequency, and courses of treatment; and (iv) Outcome measures of interest and data on outcome measures: response rate, EDSS score, FSS score, and BI of ADL.

### 2.5 Data synthesis

This study employed a Bayesian NMA approach, utilizing RStudio 2024.04.2 for data synthesis and analysis. Relationships among different acupoint stimulation methods were depicted using a network diagram, where the lines connecting the nodes represented direct comparisons. The thickness of a line was proportional to the number of studies, and the size of a node was proportional to the sample size. When a closed loop existed, node analysis was implemented to test the consistency. If all *p* values exceeded 0.05, it indicated good consistency. For each outcome variable, a fixed-effects model and a random-effects model were constructed separately. The models were selected based on the heterogeneity among studies and the deviance information criterion (DIC). If the heterogeneity among studies was low (I^2^ ≤ 50%) and the difference in DIC between the fixed-effects and random-effects models < 5, results from the fixed-effects model were adopted. If I^2^ > 50% or the difference in DIC between the two models ≥ 5, results from the random-effects model were adopted. In addition, EDSS score was treated as a continuous variable and the mean difference (MD) was used for the pooled analysis. Although EDSS score is an ordinal, non-linear outcome measure, this approach has been widely used in systematic reviews and meta-analyses on MS and is clinically interpretable (19–21). While this approximation typically does not introduce significant bias when the number of classification levels is sufficient and the data distribution is approximately symmetric, we acknowledge that it may introduce potential bias in effect estimates. The relevant limitations are further elaborated in the Discussion section.

Based on the NMA results, league tables were constructed to compare the effect sizes of the interventions, and the surface under the cumulative ranking curve (SUCRA) was used to rank the relative efficacy of different acupoint stimulation protocols.

## 3 Results

### 3.1 Results of literature search

**Figure 1:**
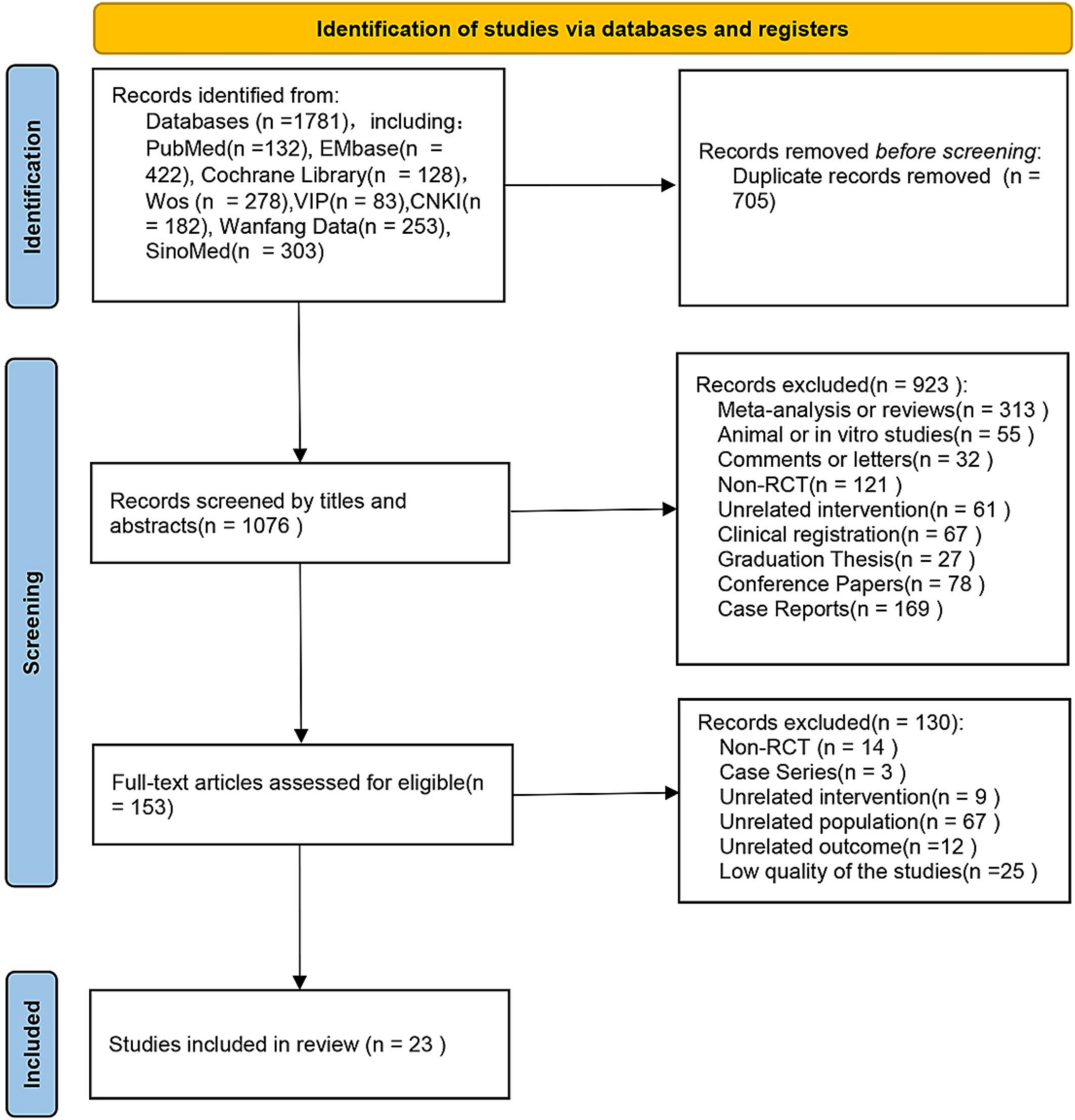
PRISMA flowchart.

Initially, 1,781 relevant articles were identified from PubMed (*n* = 132), Embase (*n* = 422), Cochrane Library (*n* = 128), Web of Science (*n* = 278), VIP (*n* = 83), SinoMed (*n* = 303), Wanfang Data (*n* = 253), and CNKI (*n* = 182). After deduplication, 705 duplicate articles were eliminated. Upon reviewing titles and abstracts, another 923 articles were excluded for failing to meet the predefined criteria. After full-text review of the remaining studies, 130 articles were excluded, including 14 articles due to non-RCT design, 3 because they were case series, 9 due to ineligible interventions, 67 due to ineligible study populations, 12 due to ineligible outcome measures, and 25 due to low quality. Ultimately, 23 RCTs were incorporated. The literature screening process is depicted in the PRISMA flowchart (Figure 1).

### 3.2 Basic characteristics of the incorporated studies

A total of 23 RCTs (11, 12, 22–42) were incorporated, involving 1,384 patients from 5 countries. Among these publications, 8 were published in English and 15 in Chinese. The 23 RCTs involved 8 distinct acupoint stimulation interventions, including 4 single interventions (acupuncture, electroacupuncture, moxibustion, and acupressure) and 4 combined interventions (Chinese herbal medicine + acupuncture, acupuncture + warm needling + plum-blossom needle, electroacupuncture + acupoint injection, and acupuncture + acupressure). The total treatment duration ranged from 2 weeks to 6 months, and the total number of treatment sessions varied from 6 to 90. Intervention frequency ranged from once weekly to once daily. Frequently used acupoints included SP 6 (*Sanyinjiao*), ST 36 (*Zusanli*), GV 20 (*Baihui*), and LI 4 (*Hegu*). Detailed application frequencies of acupoints are provided in Supplementary Table 4. The baseline characteristics of the incorporated studies are listed in Table 1.

**Table 1:** Basic information of incorporated studies.

Study ID	Study location	MS Type	Ages (T/C)	Number of participants (T/C)	Sex Ratio (male%)	Disease Duration (SD)	Intervention (T/C)	Acupoints	Duration per session (minutes)	Frequency	Duration	Outcomes
Xu 2011 (24)	China	Not specified	33.52 ± 10.21	38	52.63	4.6 ± 3.7	Chinese Herbal Medicine + Acupuncture + C	GV20, GB20, TE5, LI4, PC6, ST36, SP6, KI1, BL18, BL23	20	1 session/day	3 months	RR, EDSS
			31.73 ± 11.26	35	45.71	4.4 ± 3.2	Standard treatment					
Hu 2010 (25)	China	Not specified	43.6 ± 8.8	35	45.57	4.6 ± 3.7	Chinese Herbal Medicine + Acupuncture + C	GV20, GB20, TE5, LI4, PC6, ST36, SP6, BL18, BL23, LI11, ST40, ST41, LR3	20	1 session/every other day	3onths	EDSS
			45.7 ± 8.2	35	42.86	4.1 ± 3.2	Standard treatment					
Cui 2013 (26)	China	Not specified	35.56 ± 7.24	32	56.25	7.12 ± 3.26	Chinese Herbal Medicine + Acupuncture + C	GV20, EX-B2, HN3, GB20, ST36, LR3, GB8, GB15, LI11	30	1 session/every other day	3 months	Barthel
			33.15 ± 6.28	28	53.57	6.98 ± 2.95	Standard treatment					
Wang 2017 (27)	China	RRMS	36 ± 5	21	28.57	4.8 ± 1.9	Acupuncture + Standard treatment	CV13, CV12, CV10, CV6, ST25, ST36, PC6, GV20, GV16, GV1-14, LI11, LI4, GB34, SP6	30	5 sessions/week	6 weeks	EDSS
			34 ± 6	21	28.57	5.1 ± 2.2	Sham Stimulation + Standard treatment					
Li 2016 (28)	China	RRMS	16—60	11	NA	4.91 ± 2.74	Acupuncture + Standard treatment	G20, ST36, SP6	30	1 session/day	10 days	EDSS
			16—60	10	NA	7.35 ± 5.86	Sham Stimulation + Standard treatment					
Li 2013 (29)	China	Not specified	37.6 ± 12.8	24	37.5	NA	Acupuncture + Electroacupuncture + C	GV20, GB20, ST36, SP6, KI3, BL18-21, BL23	20	6 sessions/week	3 weeks	RR, EDSS
			38.2 ± 15.8	25	28	NA	Standard treatment					
Zhou 2017 (30)	China	Not specified	NA	32	NA	NA	Moxibustion + C	PC6, ST36, SP6, CV12, EX-B2, CV4, LU5	20	6 sessions/week	3 weeks	RR, EDSS, Barthel
			NA	32	NA	NA	Standard treatment					
Ding 2013 (31)	China		36 ± 10	20	NA	2.8 ± 0.8	Acupoint injection + C	EX-B2	NA	1 session/day	6 weeks	EDSS, Barthel
			32 ± 13	20	NA	2.4 ± 0.5	Electroacupuncture + Standard treatment	EX-B2	30	5 sessions/week	6 weeks	
Li 2020 (32)	China	RRMS	38.52 ± 8.25	23	47.83	NA	Electroacupuncture + Standard treatment	GB34, SP6, LI4, ST36, ST31, ST32, LI15, LI11EX-B2	20	5 sessions/week	6 months	RR, EDSS, Barthel
			36.61 ± 8.82	23	52.17	NA	Acupuncture + Standard treatment	GB34, SP6, LI4, ST36, ST31, ST32, LI15, LI11, EX-B2	20	5 sessions/week	6 months	
Yang 2014 (33)	China	RRMS	45.68 ± 11.98	19	36.84	4.4 ± 1.5	Acupuncture + C	PC6, SP6, LU5, HT1, GB20, BL10	30	1 session/day	28 days	EDSS, FSS
			42.45 ± 14.34	17	41.18	4.2 ± 1.8	Standard treatment					
Zheng 2013 (34)	China	Not specified	NA	28	NA	NA	Acupuncture + C	GV3-GV14, BL15-BL23, MS6, MS7, SI3, BL62, GB41, GB34, ST31, GV15, GB30, BL 40, ST36, SP6, BL60	30	1 session/day	15 days	RR
			NA	28	NA	NA	Standard treatment					
Luo 2015 (35)	China	Not specified	31.6 ± 3.4	30	36.67	2.3 ± 0.7	Acupoint injection + C	EX-B2	NA	5 sessions/week	6 months	EDSS, Barthel
			32.1 ± 3.5	30	40	2.2 ± 0.9	Electroacupuncture + Standard treatment	EX-B2	30	5 sessions/week	6 months	
Wang 2016 (36)	China	RRMS	38.56 ± 7.24	38	34.21	6.12 ± 2.46	Acupuncture + C	MS6, MS7, EX-B2 (T1-T7), ST40, SP10, GV20, GV26, LR3, LI4, HT7, PC6	30	5 sessions/week	2 months	Barthel
			36.15 ± 6.28	40	45	6.98 ± 3.15	Standard treatment					
Wu 2015 (37)	China	Not specified	33.9 ± 3.4	51	52.94	NA	Acupuncture + Acupressure + C	EX-HN 3, GB 8, GB 20, GB 15, GV 20, ST 36, LI 11, LR 3, GV3-GV14	20	1 session/every other day	6 months	RR
			34.2 ± 3.5	51	54.90	NA	Standard treatment					
Ran 2018 (38)	China	Not specified	35 ± 10.2	27	48.15	3.5 ± 3.8	Acupuncture + Warm Acupuncture + Plum blossom needle + C	①: BL10, EX-B2 (T1-L5), GB31, ST35, GB34, GB39, KI3, BL60 ②: GV20, ST36, SP6 ③: GV2-15, BL1-LL, BL2-LL	NA	1 session/day	3 months	RR, EDSS
			34 ± 10.1	24	45.83	3.4 ± 3.0	Standard treatment					
Cabanillas 2012 (11)	Brazil	RRMS	36.0 ± 11.5	16	12.5	7.6 ± 6.0	Electroacupuncture + Standard treatment	LI4, ST36, SP6, EX-HN3, LI11	30	1 session/day	6 months	EDSS
			40.1 ± 9.1	15	13.33	9.3 ± 7.0	Sham Stimulation + Standard treatment					
Yeni 2022 (22)	Turkey	Not specified	40.56 ± 10.83	41	36.59	9.43 ± 7.67	Acupressure + C	LI4, ST36, SP6	18	1 session/day	4 weeks	FSS
			42.07 ± 10.18	40	277.5	8.90 ± 5.88	Sham Stimulation + C					
			40.95 ± 11.30	42	33.33	9.22 ± 6.80	Standard treatment					
Rahimi 2020 (39)	Iran	RRMS	32.86	44	25	NA	Acupressure + C	EX-HN3, HT7	15	1 session/day	30 days	FSS
			32.18	42	28.57	NA	Sham Stimulation + C					
Khodaie 2024 (55)	Iran	RRMS	38.10 ± 7.35	31	19.35	8.87 ± 4.11	Acupuncture + Standard treatment	GV20, EX-HN1, GV24, EX-HN3, GB20, CV12, CV6, ST25, HT7, PC6, ST36, SP6, LR3	30	2 sessions/week	12 weeks	FSS
			40.16 ± 9.26	31	19.35	9.65 ± 6.35	Sham Stimulation + Standard treatment					
Khodaie 2023 (40)	Iran	RRMS	39.9 ± 9.55	30	16.67	9.0 ± 6.72	Acupuncture + C	CV4, CV6, LI4, GB34, ST36, SP10, SP6, LR3, KI3	20	2 or 3 sessions/week	4 weeks	FSS
			36.2 ± 9.47	30	16.67	6.4 ± 7.50	Standard treatment					
Bastani 2015 (41)	Iran	Not specified	31.88 ± 6.21	50	0	2.86 ± 1.27	Acupressure+ Standard treatment	ST36, SP6, LI4	18	2 sessions/day	2 weeks	FSS
			31.90 ± 6.33	50	0	3.16 ± 1.18	Sham Stimulation+ Standard treatment					
Donnellan 2008 (42)	UK	SPMS	53 ± 9	7	28.57	NA	Acupressure+ Standard treatment	Treatment Based on Pattern Differentiation	25	2 sessions/day	5 weeks	FSS
			50 ± 8	7	28.57	NA	Sham Stimulation+ Standard treatment					
Sungur 2023 (12)	Turkey	Not specified	35.65	30	30	NA	Acupressure + C	ST36, SP6, LI4	NA	3 sessions/week	4 weeks	FSS

* C, Control group intervention; RRMS, relapsing–remitting multiple sclerosis; SPMS, secondary progressive multiple sclerosis; SCMS, spinal cord multiple sclerosis; RR, Response Rate; BI, Barthel index.

### 3.3 Risk of bias assessment

Ultimately, 7 studies (30.43%) were rated “Good,” 9 studies (39.13%) were rated “Fair,” and 7 studies (30.43%) were rated “Poor.” The vast majority of studies performed well in baseline similarity, control of loss to follow-up, validity and reliability of outcome measurements, and intention-to-treat analysis. Regarding randomization, 12 studies reported specific methods such as computer-generated randomization or the random number table method. Due to the particularity of the interventions, blinding was difficult to implement. Only 5 studies reported adequate blinding of participants and intervention providers. Allocation concealment and sample size estimation may also introduce selection bias. Overall, the quality of evidence was moderate, but some risk of bias was present. The risk of bias assessment for incorporated RCTs is outlined in Supplementary Table 5.

### 3.4 NMA

#### 3.4.1 EDSS

**Figure 2:**
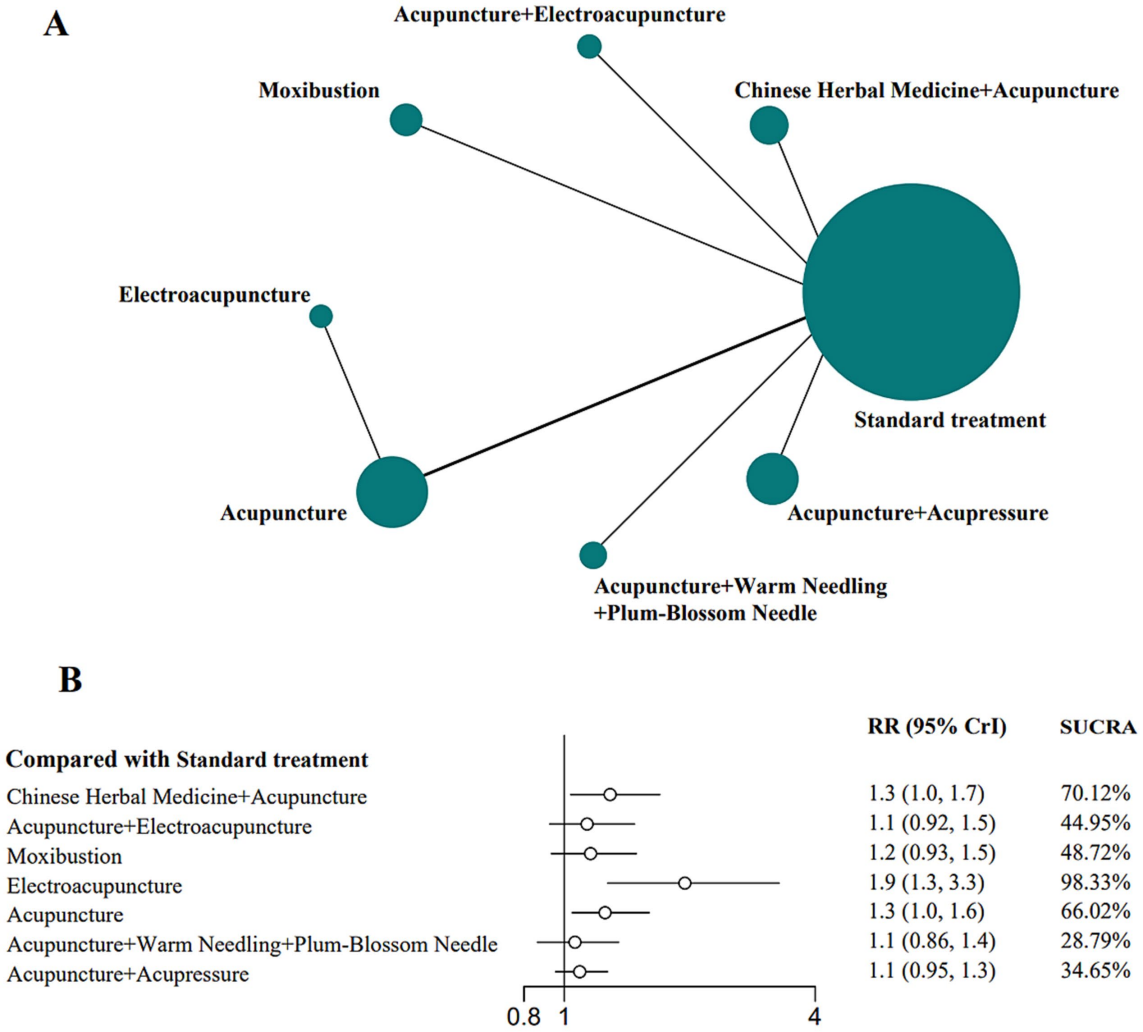
**(A)** Network diagram for EDSS; **(B)** Forest plot and SUCRA values for EDSS.

EDSS was reported as an outcome measure in 12 studies involving 583 patients, which evaluated 9 interventions (network diagram shown in Figure 2A). Among the 9 direct comparisons, 2 exhibited marked heterogeneity (I^2^ > 50%), with no overall inconsistency observed (*p* > 0.05) (Supplementary Figure 1). Given the minimal overall heterogeneity (I^2^ = 7%) and a DIC difference of 0.96 between the random-effects and fixed-effects models, the fixed-effects model was selected. The forest plot for relative effects indicated that, compared with standard treatment alone, electroacupuncture combined with acupoint injection (MD = −2.9, 95% CrI: −3.4 to −2.3), electroacupuncture (MD = −1.5, 95% CrI: −1.8 to −1.1), moxibustion (MD = −1.1, 95% CrI: −2.0 to −0.23), Chinese herbal medicine combined with acupuncture (MD = −0.87, 95% CrI: −1.3 to −0.40), and acupuncture alone (MD = −0.24, 95% CrI: −0.40 to −0.091) all reduced EDSS scores. Among these, electroacupuncture + acupoint injection was the most effective. SUCRA probability ranking indicated that electroacupuncture combined with acupoint injection was most likely to be the optimal adjuvant intervention (SUCRA = 99.99%), followed by electroacupuncture alone (SUCRA = 81.69%). The forest plot for relative effects and SUCRA values are shown in Figure 2B. The results of pairwise comparisons are listed in Table 2.

**Table 2:** League table for EDSS.

Standard treatment								
**0.87 (0.4, 1.33)**	Chinese herbal medicine+Acupuncture							
0.16 (−0.82, 1.13)	−0.71 (−1.79, 0.37)	Acupuncture+Electroacupuncture						
**1.12 (0.23, 2.01)**	0.25 (−0.75, 1.25)	0.96 (−0.36, 2.28)	Moxibustion					
**1.45 (1.12, 1.78)**	**0.58 (0.01, 1.16)**	**1.3 (0.27, 2.33)**	0.33 (−0.61, 1.28)	Electroacupuncture				
**0.24 (0.09, 0.4)**	**−0.63 (−1.11, −0.13)**	0.09 (−0.9, 1.08)	−0.88 (−1.77, 0.03)	**−1.21 (−1.55, −0.86)**	Acupuncture			
0.7 (−0.3, 1.7)	−0.17 (−1.26, 0.94)	0.54 (−0.84, 1.95)	−0.42 (−1.75, 0.93)	−0.75 (−1.8, 0.3)	0.45 (−0.56, 1.47)	Acupuncture+Warm Needling+Plum-Blossom Needle		
**2.89 (2.34, 3.44)**	**2.02 (1.29, 2.75)**	**2.74 (1.61, 3.85)**	**1.77 (0.72, 2.82)**	**1.44 (1, 1.88)**	**2.65 (2.08, 3.21)**	**2.2 (1.05, 3.34)**	Electroacupuncture	
0.73 (−0.34, 1.81)	−0.13 (−1.32, 1.04)	0.58 (−0.87, 2.03)	−0.39 (−1.79, 1.02)	−0.72 (−1.84, 0.41)	0.49 (−0.58, 1.55)	0.03 (−1.43, 1.5)	−2.16 (−3.37, −0.95)	Sham Stimulation

Bold values indicate statistically significant differences (95% CrI does not include 0).

#### 3.4.2 FSS

**Figure 3:**
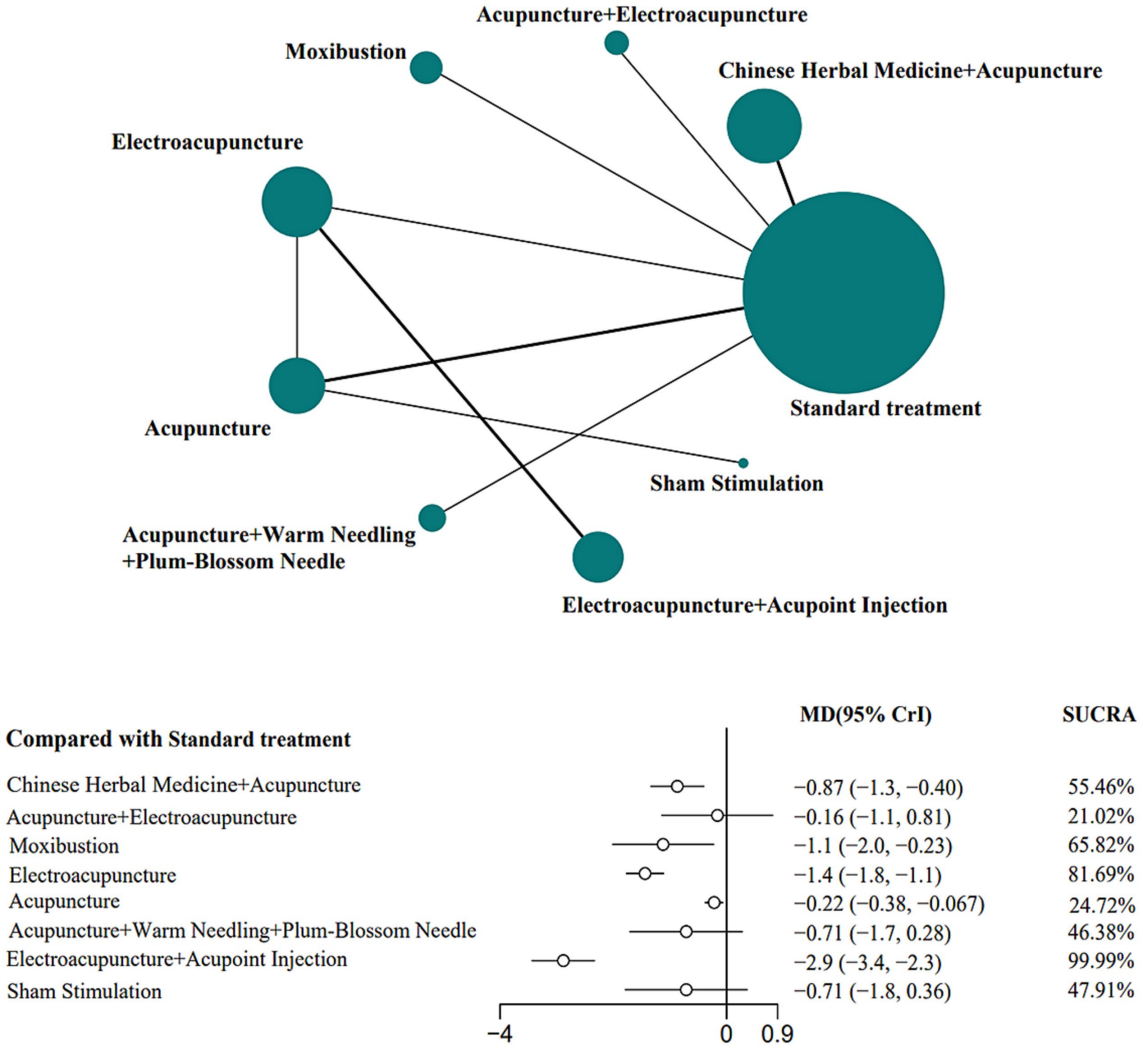
**(A)** Network diagram for FSS; **(B)** Forest plot and SUCRA values for FSS.

FSS was reported as an outcome measure in 8 studies involving 541 participants across 4 interventions (network diagram shown in Figure 3A). Among the 5 direct comparisons, 2 exhibited substantial heterogeneity (I^2^ > 50%). No statistical evidence of global or local inconsistency was found (*p* > 0.05) (Supplementary Figure 2). Given the minimal overall heterogeneity (I^2^ = 5%) and a DIC difference of 1.09 between the random-effects and fixed-effects models, the fixed-effects model was selected. The forest plot for relative effects indicated that, compared with standard treatment alone, acupressure (standardized mean difference [SMD] = −1.3, 95% CrI: −1.6 to −1.0), acupuncture (SMD = −0.88, 95% CrI: −1.2 to −0.59) and sham stimulation (SMD = −0.56, 95% CrI: −0.89 to −0.24) all reduced patients’ FSS scores. SUCRA probability ranking indicated that acupressure may be the most effective adjuvant therapy for reducing FSS scores (SUCRA = 99.27%), followed by acupuncture (SUCRA = 65.19%) and sham stimulation (SUCRA = 35.52%). The forest plot for relative effects and SUCRA values are shown in Figure 3B. The results of pairwise comparisons are listed in Table 3.

**Table 3:** League table for FSS.

Standard treatment			
0.88 (0.59, 1.18)	Acupuncture		
1.29 (1, 1.59)	0.42 (0.01, 0.82)	Acupressure	
0.56 (0.24, 0.89)	−0.32 (−0.73, 0.1)	−0.73 (−0.96, −0.5)	Sham Stimulation

#### 3.4.3 BI

**Figure 4:**
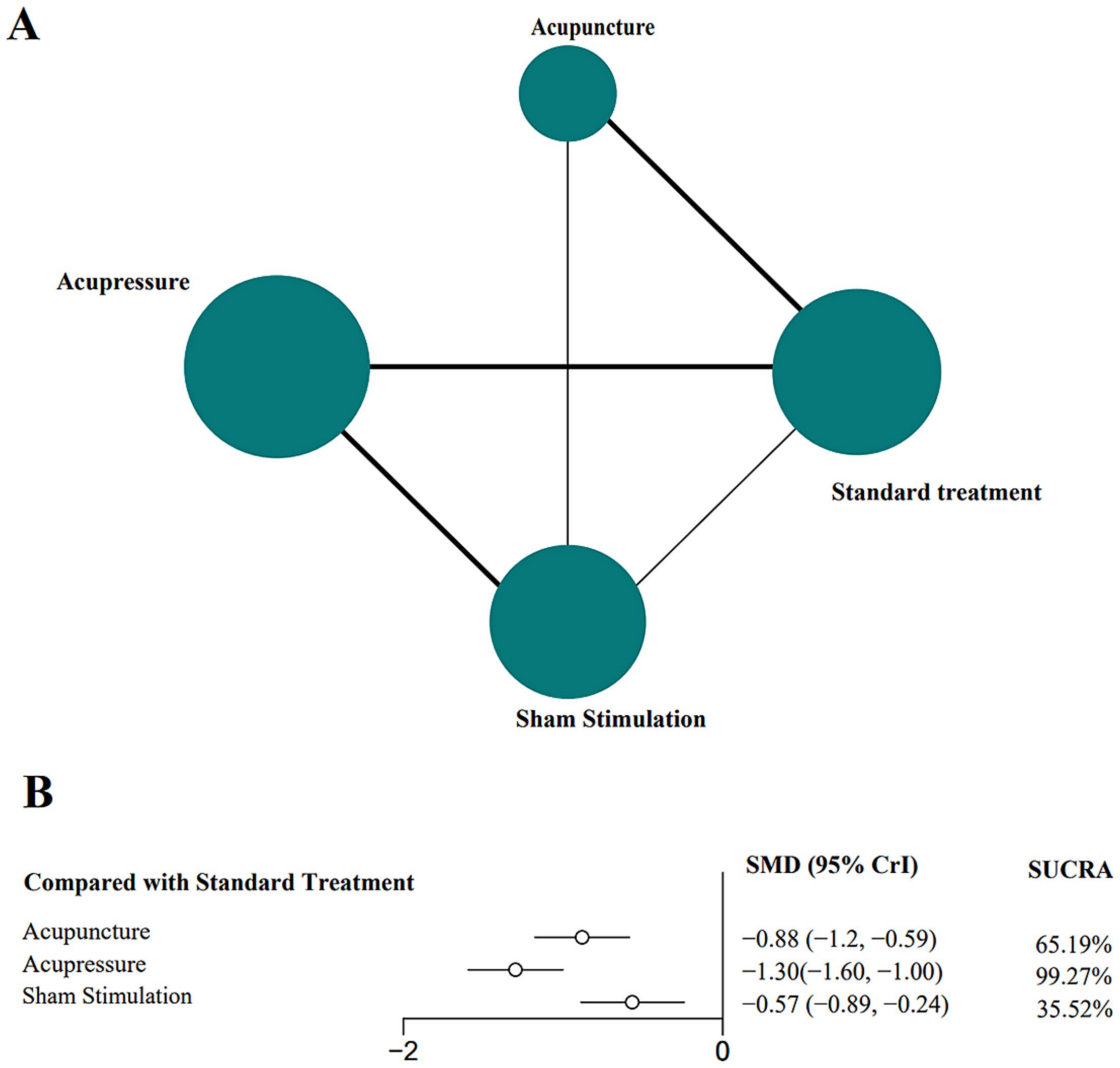
**(A)** Network diagram for BI; **(B)** Forest plot and SUCRA values for BI.

BI for ADL was reported as an outcome measure in 6 studies involving 348 participants across 6 interventions (network diagram shown in Figure 4A). No substantial heterogeneity was observed in any of the 5 direct comparisons (Supplementary Figure 3). Given the minimal overall heterogeneity (I^2^ = 5%) and a DIC difference of 1.09 between the random-effects and fixed-effects models, the fixed-effects model was selected. The forest plot for relative effects indicated that, compared with standard treatment alone, electroacupuncture combined with acupoint injection (MD = 18, 95% CrI: 8.8 to 28) markedly improved patients’ BI. Acupuncture (MD = 5.5, 95% CrI: 1.3 to 9.8) and moxibustion (MD = 7.1, 95% CrI: 1.0 to 13) also improved the response rate to MS treatment. SUCRA probability ranking indicated that electroacupuncture + acupoint injection showed the most significant improvement in BI (SUCRA = 98.75%), followed by electroacupuncture alone (SUCRA = 61.12%). The forest plot for relative effects and SUCRA values are shown in Figure 4B. The results of pairwise comparisons are listed in Table 4.

**Table 4:** League table for BI.

Standard treatment					
−3.91 (−16.45, 8.7)	Chinese herbal medicine + Acupuncture				
−7.05 (−12.96, −1.03)	−3.15 (−17.02, 10.75)	Moxibustion			
−8.46 (−17.45, 0.55)	−4.48 (−20.01, 10.84)	−1.43 (−12.21, 9.35)	Electroacupuncture		
−5.5 (−9.76, −1.27)	−1.56 (−14.92, 11.72)	1.55 (−5.79, 8.81)	2.95 (−4.96, 10.86)	Acupuncture	
−18.22 (−27.63, −8.8)	−14.26 (−30.04, 1.33)	−11.16 (−22.34, −0.08)	−9.76 (−12.47, −7)	−12.72 (−21.13, −4.31)	Electroacupuncture + Acupoint Injection

#### 3.4.4 Response rate

**Figure 5:**
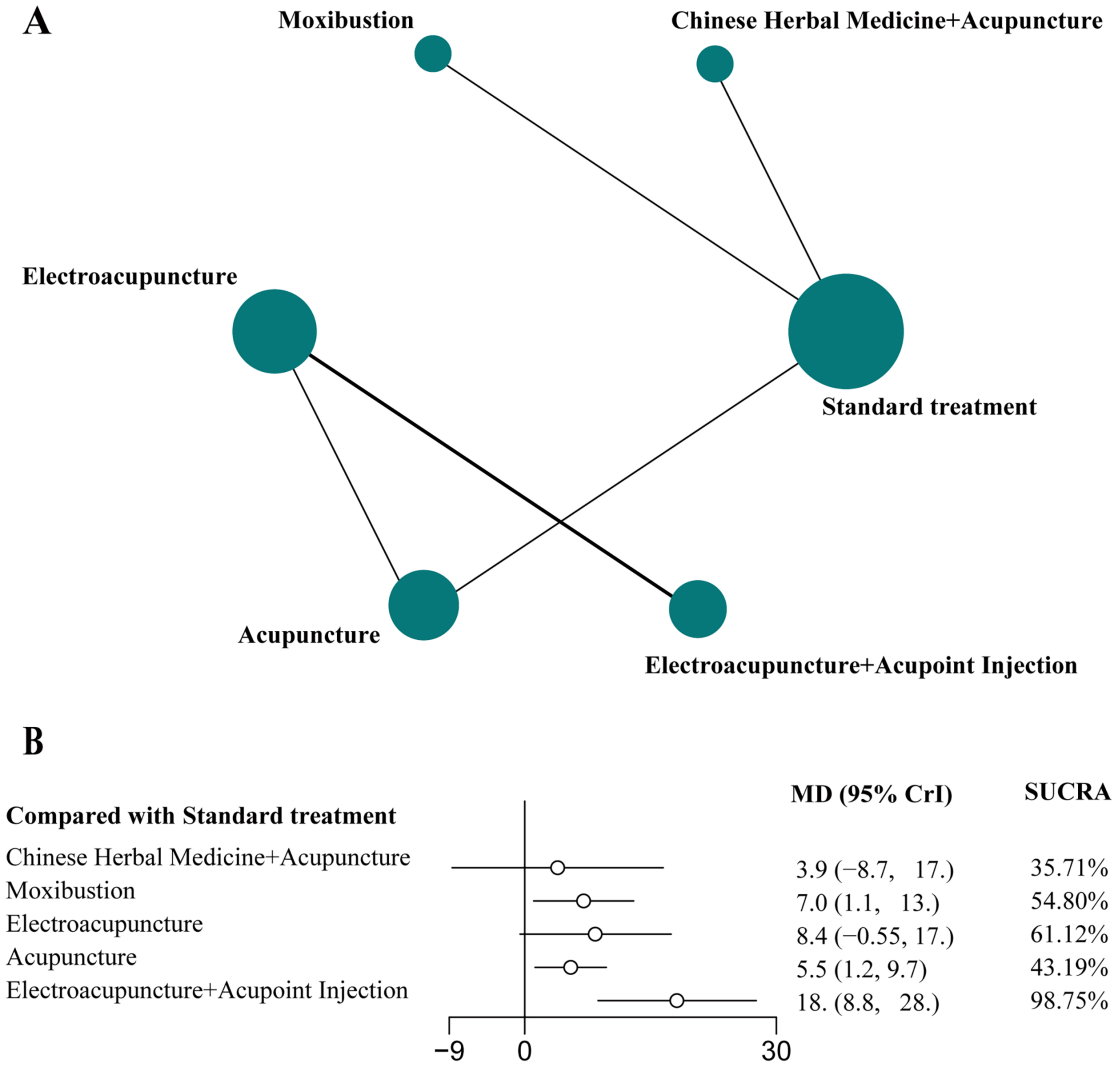
**(A)** Network diagram for response rate; **(B)** Forest plot and SUCRA values for response rate.

Due to variations in the definition of response rate across the included studies, and given that this indicator is not a recognized primary endpoint in research on MS, the following results are presented solely as an exploratory analysis and should be interpreted with caution. Response rate was reported in 8 studies, involving 477 patients across 8 interventions (network diagram shown in Figure 5A). No substantial heterogeneity was detected in the 7 direct comparisons, as illustrated in Supplementary Figure 4. Given the small overall heterogeneity (I^2^ = 18%) and a DIC difference of 0.6 between random-effects and fixed-effects models, the fixed-effects model was selected. The forest plot for relative effects indicated that, compared with standard treatment, electroacupuncture markedly enhanced the response rate to MS treatment (risk ratio [RR] = 1.9, 95% credible interval [CrI]: 1.3 to 3.3). Acupuncture therapy (RR = 1.3, 95% CrI: 1.0 to 1.6) and Chinese herbal medicine + acupuncture therapy (RR = 1.3, 95% CrI: 1.0 to 1.7) also increased the response rate to MS treatment. SUCRA probability ranking indicated that standard treatment combined with electroacupuncture was most likely to be the optimal intervention for improving the response rate to MS treatment (SUCRA = 98.33%), followed by Chinese herbal medicine combined with acupuncture (SUCRA = 70.12%). The forest plot for relative effects and SUCRA values are shown in Figure 5B. The results of pairwise comparisons are listed in Table 5.

**Table 5:** League table for response rate.

Standard treatment							
**0.77 (0.59, 0.96)**	Chinese herbal medicine+Acupuncture						
**0.88 (0.68, 1.08)**	**1.13 (0.81, 1.6)**	Acupuncture+Electroacupuncture					
**0.86 (0.67, 1.07)**	**1.12 (0.79, 1.59)**	**0.98 (0.71, 1.37)**	Moxibustion				
**0.51 (0.3, 0.79)**	**0.67 (0.38, 1.11)**	**0.59 (0.33, 0.96)**	**0.6 (0.34, 0.98)**	Electroacupuncture			
**0.8 (0.62, 0.96)**	**1.03 (0.74, 1.44)**	**0.91 (0.66, 1.25)**	**0.92 (0.67, 1.27)**	**1.54 (1.06, 2.48)**	Acupuncture		
**0.94 (0.74, 1.16)**	**1.22 (0.89, 1.72)**	**1.07 (0.79, 1.49)**	**1.09 (0.79, 1.52)**	**1.84 (1.13, 3.21)**	**1.18 (0.88, 1.63)**	Acupuncture+Warm Needling+Plum-Blossom Needle	
**0.92 (0.79, 1.05)**	**1.19 (0.91, 1.61)**	**1.04 (0.81, 1.39)**	**1.06 (0.82, 1.42)**	**1.78 (1.13, 3.05)**	**1.15 (0.91, 1.52)**	**0.97 (0.75, 1.28)**	Acupuncture+Acupressure

Bold values indicate statistically significant differences (95% CrI does not include 0).

## 4 Discussion

This study systematically evaluated 23 RCTs involving 1,384 patients with MS and 8 acupoint stimulation interventions. The outcome measures covered 3 key domains: overall neurological function (EDSS), fatigue (FSS), and ADL (BI). The relative efficacy of various acupoint stimulation therapies as adjuncts to standard treatment was compared. Overall, the combination of acupuncture stimulation therapy with standard treatment showed a trend toward positive improvement to varying degrees across all outcome measures.

According to the European Committee of Treatment and Research in Multiple Sclerosis (ECTRIMS) and the European Academy of Neurology (EAN) guidelines (7), the comprehensive management of MS comprises three major approaches: DMT, acute relapse management, and symptomatic and supportive care (43, 44). The reference intervention in this study, namely standard treatment, encompassed medications such as fingolimod, interferon beta, and methylprednisolone. However, current guidelines provide limited evidence and lower-level recommendations for non-pharmacological interventions targeting symptoms such as fatigue and neurological deficits, leaving unmet clinical needs. Acupoint stimulation therapy, as a complementary and alternative therapy (CAT), holds considerable potential for symptomatic supportive care and rehabilitation management in MS.

In this study, acupuncture therapy demonstrated varying degrees of improvement in EDSS, FSS, and BI outcomes, which largely aligned with the results of the meta-analysis by Chen et al. (45). However, it should be noted that although the reduction in EDSS scores associated with acupuncture in this study (MD = −0.24) was statistically significant, it did not reach the generally accepted threshold for clinical significance (46, 47), suggesting that its clinical value requires to be further validated. Electroacupuncture is a therapeutic method in which micro-pulse currents resembling the human body’s bioelectricity are applied in addition to acupuncture. The results of this study indicated that electroacupuncture reduced EDSS scores and improved BI scores. The findings of Haider et al. (48) also support the efficacy of both acupuncture and electroacupuncture in alleviating fatigue and improving quality of life. In the two studies included in this review that involved electroacupuncture combined with acupoint injection, the reduction in EDSS scores in the observation groups was significant. This may be attributed to the patients’ high baseline degree of disability at enrollment, as well as the timely initiation of electroacupuncture combined with acupoint injection following steroid pulse therapy during the acute phase, which capitalized on the critical window for neural functional repair. However, as this evidence is derived solely from two small-scale studies, caution is warranted in interpreting these results. Acupressure showed good efficacy in alleviating fatigue in MS patients, consistent with the results of the NMA by Chang et al. (49). Sham stimulation also achieved relatively satisfactory results in alleviating fatigue. Among the 8 included studies evaluating the FSS scores, 5 included a sham stimulation control group and all employed a blinded design. The results showed that the combination regimen with sham stimulation was still associated with reduced FSS scores. Since the FSS is a subjective assessment tool, this finding suggests that even with relatively rigorous blinding, the placebo effect may still exert a considerable influence on fatigue-related outcomes. Therefore, when interpreting the study results, it is necessary to acknowledge that the observed efficacy includes a certain degree of nonspecific effects, and that the true effect may be lower than the observed point estimates.

To date, some basic studies have explored the potential mechanisms by which acupoint stimulation therapy alleviates symptoms in MS patients. Regarding acupuncture, previous research suggests that it may exert effects by modulating immune responses, reducing inflammatory responses, and promoting myelin regeneration and repair (50). Electroacupuncture may enhance therapeutic efficacy by maintaining uniform and sustained stimulation intensity, while low-frequency pulsed electromagnetic fields could potentially facilitate nerve impulse conduction and neural stem cell proliferation, thereby accelerating myelin repair (51, 52). The two studies included in this review that involved acupoint injection both used mouse nerve growth factor (mNGF) for injection. Exogenous nerve growth factor supports the survival of damaged neurons and promotes synaptic growth (53), which may partially explain the efficacy of this combination therapy in improving patients’ neurological function and quality of life. Furthermore, a neuroimaging study has suggested that acupressure may reduce patients’ perception of fatigue by modulating the connectivity between the posterior insula and the dorsolateral prefrontal cortex, or by influencing the functional connectivity between the default mode network and the thalamus (54).

Furthermore, although the BI is a classic tool for assessing ADL and has demonstrated good reliability and validity in research on MS, it adopts a four-grade rating scale and coarse classification for items. Besides, the functional impairments in MS patients are complex and heterogeneous. Therefore, using the BI alone may not comprehensively and sensitively reflect subtle changes in patients’ neurological function.

This study also analyzed the response rate as an exploratory indicator. However, there is a lack of uniform criteria for determining the response rate across the 8 included studies. Besides, this indicator is not a widely recognized primary endpoint in clinical trials for MS. Therefore, although the NMA in this study indicated that interventions such as electroacupuncture were associated with a favorable trend for this indicator, these findings should be interpreted with caution and should not be over-generalized.

Although the interventions included in this study are all grounded in the meridian theory of TCM and target acupoints, they exhibit significant differences in terms of operation mode, stimulation intensity, and physiological mechanisms. These differences may act as unmeasured effect modifiers, thereby introducing bias into the results of indirect comparisons. Therefore, the findings of this study regarding the application potential of acupoint stimulation therapy in the treatment of MS, namely, that combining acupoint stimulation with standard treatment may yield additional benefits in improving neurological function, alleviating fatigue, and enhancing quality of life, should be interpreted as exploratory, hypothesis-generating evidence rather than definitive conclusions. Acupoint stimulation therapy cannot replace first-line standard treatment but may be considered as a CAT in the following scenarios: for patients with fatigue as the primary symptom who respond poorly to conventional treatment or cannot tolerate side effects; for patients who wish to add non-pharmacological interventions to standard treatment to improve quality of life; and for long-term symptom management and rehabilitation during the remission phase. Regarding specific treatment options, electroacupuncture or electroacupuncture combined with acupoint injection may be considered for those aiming to improve neurological function and activities of daily living. Acupressure is easy to perform, cost-effective, and associated with minimal side effects, which can be self-administered by trained patients, making it highly feasible for the long-term management of fatigue.

Adhering to rigorous methodology, this study systematically reviewed currently available clinical studies to synthesize evidence across three core dimensions of MS: neurological function (EDSS score), degree of fatigue (FSS score), and quality of life (BI for ADL). It provides an objective and comprehensive evaluation of the efficacy of acupoint stimulation in improving the neurological function and quality of life in patients with MS. However, some limitations exist. In this study, the EDSS score is treated as a continuous variable in the pooled analysis. Although this approach is widely accepted in this field and facilitates clinical interpretation, it may still introduce bias into the effect estimation. Due to the characteristics of acupoint stimulation therapy, it is difficult to blind investigators and participants. This is particularly true when subjective measures such as the FSS scale are analyzed, and may have a certain impact on the reliability of the results. Therefore, the observed effect sizes for all outcome measures based on subjective scales should be interpreted with caution. After excluding 7 low-quality studies (30.43%), the remaining evidence could not form a complete network. Therefore, sensitivity analysis based on network connectivity could not be conducted. For subjective scales such as the FSS, low-quality studies are more prone to overestimating the efficacy of interventions, which may lead to inflated rankings for certain interventions. Therefore, these results should be interpreted with caution. Additionally, there are significant differences in the choice of drugs, dosages, and treatment durations for the standard treatment across the included studies. This pharmacological heterogeneity may violate the transitivity assumption of NMA. Regarding safety, most included studies fail to systematically report adverse events, making it impossible to effectively compare and evaluate the safety of different acupoint stimulation regimens. This gap in evidence must be considered when translating the findings into clinical recommendations. Finally, the included studies are primarily from China and some Middle Eastern countries, representing a concentrated regional distribution. This may limit the generalizability of the findings due to racial, medical, and cultural differences. Future multicenter, transnational studies are warranted to validate the applicability of these findings.

## 5 Conclusion

This study suggests that using acupoint stimulation therapy as an adjunct to standard pharmacological treatment may hold potential value for alleviating fatigue, improving neurological function, and enhancing quality of life in patients with MS. Among these therapies, electroacupuncture, electroacupuncture combined with acupoint injection, and acupressure showed particularly prominent trends in efficacy. These interventions could be prioritized for validation in future prospective studies. However, due to limitations such as clinical heterogeneity of the interventions, difficulties in implementing blinding (which may introduce placebo effects, particularly in subjective outcome measures), and the heterogeneity in outcome measures, the results of this study are exploratory in nature and should not be directly translated into routine clinical recommendations. Future studies should include rigorously designed, well-reported multicenter RCTs to rigorously validate the efficacy and safety of acupoint stimulation therapy for the treatment of MS.

## Data Availability

The original contributions presented in the study are included in the article/Supplementary material, further inquiries can be directed to the corresponding author.

## Footnotes

1
https://www.nhlbi.nih.gov/health-topics/study-quality-assessment-tools

## References

[1] Montalban X Lebrun-Frénay C Oh J Arrambide G Moccia M Pia Amato M . Diagnosis of multiple sclerosis: 2024 revisions of the McDonald criteria. Lancet Neurol. (2025) 24:850–65. doi: 10.1016/S1474-4422(25)00270-4, 40975101

[2] Grosu C Ignat EB Alexa D Ciubotaru A Leon MM Maștaleru A . The role of nutrition and physical activity in modulating disease progression and quality of life in multiple sclerosis. Nutrients. (2025) 17:2713. doi: 10.3390/nu17162713, 40871741 PMC12389322

[3] Wang M Liu C Zou M Niu Z Zhu J Jin T. Recent progress in epidemiology, clinical features, and therapy of multiple sclerosis in China. Ther Adv Neurol Disord. (2023) 16:17562864231193816. doi: 10.1177/17562864231193816, 37719665 PMC10504852

[4] Bonomi S Jin S Culpepper WJ Wallin MT. MS and disability progression in Latin America, Africa, Asia and the Middle East: a systematic review. Mult Scler Relat Disord. (2021) 51:102885. doi: 10.1016/j.msard.2021.102885, 33773273

[5] Shipley J Beharry J Yeh W Seery N Foong YC Ayton D . Consensus recommendations on multiple sclerosis management in Australia and New Zealand: part 2. Med J Aust. (2025) 222:365–71. doi: 10.5694/mja2.52577, 39923190

[6] Shipley J Beharry J Yeh W Seery N Foong YC Ayton D . Consensus recommendations on multiple sclerosis management in Australia and New Zealand: part 1. Med J Aust. (2025) 222:356–64. doi: 10.5694/mja2.52578, 39923189

[7] Montalban X Gold R Thompson AJ Otero-Romero S Amato MP Chandraratna D . ECTRIMS/EAN guideline on the pharmacological treatment of people with multiple sclerosis. Mult Scler. (2018) 24:96–120. doi: 10.1177/1352458517751049, 29353550

[8] Silbermann E Senders A Wooliscroft L Rice J Cameron M Waslo C . Cross-sectional survey of complementary and alternative medicine used in Oregon and Southwest Washington to treat multiple sclerosis: a 17-year update. Mult Scler Relat Disord. (2020) 41:102041. doi: 10.1016/j.msard.2020.102041, 32200340

[9] Lopez-Alcalde J Steinemann N Moll H Canella C Barth J Haegele-Link S . Characteristics and expectations of people with multiple sclerosis using complementary therapies: a cross-sectional survey from the swiss multiple sclerosis registry. Mult Scler Relat Disord. (2025) 96:106349. doi: 10.1016/j.msard.2025.106349, 40058157

[10] Buttolph L Bruton AM Filbin P Wexler RS Gray O Mazure T . Effects of mind-body movement interventions for managing symptoms in people with multiple sclerosis: an overview of reviews. Curr Neurol Neurosci Rep. (2026) 26:10. doi: 10.1007/s11910-025-01478-8, 41619131 PMC12860761

[11] Quispe-Cabanillas JG Damasceno A von Glehn F Brandão CO Damasceno BP Silveira WD . Impact of electroacupuncture on quality of life for patients with relapsing-remitting multiple sclerosis under treatment with immunomodulators: a randomized study. BMC Complement Altern Med. (2012) 12:209. doi: 10.1186/1472-6882-12-209, 23126260 PMC3565890

[12] Sungur M Ovayolu N Akçalı A. The effect of acupressure applied to patients with multiple sclerosis on fatigue. Holist Nurs Pract. (2023) 37:184–94. doi: 10.1097/HNP.0000000000000588 37335146

[13] Zhao J Wang L Li Y. Electroacupuncture alleviates the inflammatory response via effects on M1 and M2 macrophages after spinal cord injury. Acupunct Med. (2017) 35:224–30. doi: 10.1136/acupmed-2016-011107, 28077367

[14] Kang Z Zou ZF Sun JX Zhu KY Jiang JW Wu GC . Electroacupuncture promotes regeneration and repair of myelin sheath of corpus callosum in demyelination mice. Zhen Ci Yan Jiu. (2020) 45:1–7. doi: 10.13702/j.1000-0607.1901626, 32144901

[15] Hutton B Salanti G Caldwell DM Chaimani A Schmid CH Cameron C . The PRISMA extension statement for reporting of systematic reviews incorporating network meta-analyses of health care interventions: checklist and explanations. Ann Intern Med. (2015) 162:777–84. doi: 10.7326/M14-2385, 26030634

[16] Thompson AJ Banwell BL Barkhof F Carroll WM Coetzee T Comi G . Diagnosis of multiple sclerosis: 2017 revisions of the McDonald criteria. Lancet Neurol. (2018) 17:162–73. doi: 10.1016/S1474-4422(17)30470-2, 29275977

[17] Polman CH Reingold SC Banwell B Clanet M Cohen JA Filippi M . Diagnostic criteria for multiple sclerosis: 2010 revisions to the McDonald criteria. Ann Neurol. (2011) 69:292–302. doi: 10.1002/ana.22366, 21387374 PMC3084507

[18] Polman CH Reingold SC Edan G Filippi M Hartung HP Kappos L . Diagnostic criteria for multiple sclerosis: 2005 revisions to the "McDonald criteria". Ann Neurol. (2005) 58:840–6. doi: 10.1002/ana.20703, 16283615

[19] Pihl-Jensen G Tsakiri A Frederiksen JL. Statin treatment in multiple sclerosis: a systematic review and meta-analysis. CNS Drugs. (2015) 29:277–91. doi: 10.1007/s40263-015-0239-x, 25795002

[20] Lattanzi S Cagnetti C Danni M Provinciali L Silvestrini M. Oral and intravenous steroids for multiple sclerosis relapse: a systematic review and meta-analysis. J Neurol. (2017) 264:1697–704. doi: 10.1007/s00415-017-8505-0, 28492970

[21] Norris CM Ghali WA Saunders LD Brant R Galbraith D Faris P . Ordinal regression model and the linear regression model were superior to the logistic regression models. J Clin Epidemiol. (2006) 59:448–56. doi: 10.1016/j.jclinepi.2005.09.007, 16632132

[22] Yeni K Tulek Z Terzi M. Effect of self-acupressure on fatigue in patients with multiple sclerosis. Complement Ther Clin Pract. (2022) 47:101572. doi: 10.1016/j.ctcp.2022.101572, 35316705

[23] Khodaie F Saeedi R Soleimany G Sahraian MA Kazemi AH Moghadasi AN . Effects of acupuncture on cognitive functions in patients with relapsing-remitting multiple sclerosis: a randomized controlled trial. Chin J Integr Med. (2025) 31:928–36. doi: 10.1007/s11655-025-3814-0, 39762502

[24] Xu XM Xiao C.Y., editor Treatment of 38 cases of multiple sclerosis with integrated traditional Chinese and Western medicine. Jiangxi J Tradit Chin Med; (2011) 42:34–36.

[25] Hu YY Liu T Hu YQ He QC Zhang QP Liang N. Clinical study on 35 cases of multiple sclerosis treated with integrated traditional Chinese and Western medicine. Jiangsu J Tradit Chin Med. (2010) 42:23–4.

[26] Cui HS Chen SX Hong YF Qin LF. Clinical research on acupuncture combined with herbal medicine for multiple sclerosis. Acad J Shanghai Univ Tradit Chin Med. (2013) 27:48–50.

[27] Wang C Chen Z Wang L Ma X Xing Y Li A . Relapsing-remitting multiple sclerosis at remission stage treated with acupuncture:a randomized controlled trial. Zhongguo Zhen Jiu. (2017) 37:576–80. doi: 10.13703/j.0255-2930.2017.06.002, 29231495

[28] Li KN Fan YP Wang WM Li Q. Evaluation of the efficacy of acupuncture on fatigue in patients with relapsing-remitting multiple sclerosis and its effects on serum interleukin-1β and tumor necrosis factor-α. Glob Tradit Chin Med. (2016) 9:1024–6.

[29] Li CX. Acupuncture at back-Shu points for 24 cases of multiple sclerosis. J Clin Acupunct Moxibustion. (2013) 29:42–4.

[30] Zhou ZY Lu CJ Wang HH Wei BX Si T. Yao medicine shenhuo moxibustion combined with hormone therapy for multiple sclerosis. Jilin J Tradit Chin Med. (2017) 37:528–531+40.

[31] Ding Y Shi X. A controlled study on Electroacupuncture combined with Acupoint injection for multiple sclerosis. Chin Acupunct Moxibustion. (2013) 33:793–5. 24298767

[32] Li SY Zhang B Wu X. Electroacupuncture combined with medication for 46 cases of relapsing-remitting multiple sclerosis. Henan Tradit Chin Med. (2020) 40:1426–8.

[33] Yang ZH Li GQ Yuan B Yang LF Guo XJ. Observation on the efficacy of neurological rehabilitation in the remission phase of multiple sclerosis. J Xinxiang Med Univ. (2014) 31:57–9.

[34] Zheng P Zhang WM. Observation on the efficacy of acupuncture and herbal medicine for multiple sclerosis. China Health Care Nutr. (2013) 4:586.

[35] Luo YY. A controlled study on Electroacupuncture combined with Acupoint injection for multiple sclerosis. Pract Clin J Integr Tradit Chin West Med. (2015) 15:37–8.

[36] Wang JW Huang SH Fan SY Yu LZ Chen YP Lan PF. Clinical study on Wen Tong (warming and unblocking) acupuncture therapy for multiple sclerosis of phlegm-stasis obstruction type. New Chin Med. (2016) 48:129–31.

[37] Wu LQ Shi SY. Analysis of the clinical value of combining Tuina and acupuncture in the treatment of multiple sclerosis. Med Inf. (2015) 3:46.

[38] Ran LL Meng J Wang L. Observation on the clinical efficacy of warm needling combined with plum-blossom needling for multiple sclerosis. Hubei J Tradit Chin Med. (2018) 40:50–1.

[39] Rahimi H Mehrpooya N Vagharseyyedin S BahramiTaghanaki H. Self-acupressure for multiple sclerosis-related depression and fatigue: a feasibility randomized controlled trial. J Adv Med Biomed Res. (2020) 28:276–83. doi: 10.30699/jambs.28.130.276

[40] Khodaie F Naser Moghadasi A Kazemi AH Zhao B. Effectiveness of acupuncture for fatigue in patients with relapsing-remitting multiple sclerosis: a randomized controlled trial. Acupunct Med. (2023) 41:199–205. doi: 10.1177/09645284221150824, 36722418

[41] Bastani F Sobhani M Emamzadeh Ghasemi HS. Effect of acupressure on fatigue in women with multiple sclerosis. Glob J Health Sci. (2015) 7:375–81. doi: 10.5539/gjhs.v7n4p375, 25946938 PMC4802202

[42] Donnellan CP Shanley J. Comparison of the effect of two types of acupuncture on quality of life in secondary progressive multiple sclerosis: a preliminary single-blind randomized controlled trial. Clin Rehabil. (2008) 22:195–205. doi: 10.1177/0269215507082738, 18285429

[43] Boutitah-Benyaich I Eixarch H Villacieros-Álvarez J Hervera A Cobo-Calvo Á Montalban X . Multiple sclerosis: molecular pathogenesis and therapeutic intervention. Signal Transduct Target Ther. (2025) 10:324. doi: 10.1038/s41392-025-02415-4, 41034190 PMC12488951

[44] Pithakumar A Basha S Pai AR Mahato KK. From pathogenesis to precision medicine: targeting immune imbalance in multiple sclerosis. Ageing Res Rev. (2026) 113:102921. doi: 10.1016/j.arr.2025.102921, 41109515

[45] Chen Y Cui Y Zhou X Zhang S Wang Z Yang J . Clinical evidence on acupuncture for symptom improvement in multiple sclerosis. Complement Ther Med. (2025) 95:103276. doi: 10.1016/j.ctim.2025.103276, 41161577

[46] Montolío A Cegoñino J Garcia-Martin E Pérez Del Palomar A. Comparison of machine learning methods using Spectralis OCT for diagnosis and disability progression prognosis in multiple sclerosis. Ann Biomed Eng. (2022) 50:507–28. doi: 10.1007/s10439-022-02930-3, 35220529 PMC9001622

[47] CADTH Common Drug Reviews. Clinical Review Report: Daclizumab (Zinbryta). Ottawa (ON): Canadian Agency for Drugs and Technologies in HealthCopyright © 2017 Canadian Agency for Drugs and Technologies in Health (2017). 30561964

[48] Haider S Fatmi W Shoaib N Sajjad M Zahid M. Assessment of acupuncture's effectiveness in mitigating fatigue among patients afflicted with multiple sclerosis: a systematic review and meta-analysis. Complement Ther Clin Pract. (2024) 57:101902. doi: 10.1016/j.ctcp.2024.101902, 39260078

[49] Chang H Wang X Shi Y. Comparative efficacy of non-pharmacological interventions on fatigue in people with multiple sclerosis: a systematic review and network meta-analysis. Int J Nurs Stud. (2026) 173:105250. doi: 10.1016/j.ijnurstu.2025.105250, 41167042

[50] Tang X Wang C Tian S Wen H Zhang H. Acupuncture for neurodegenerative diseases: mechanisms, efficacy, and future research directions. Am J Transl Res. (2025) 17:3703–17. doi: 10.62347/QFJO6227, 40535632 PMC12170434

[51] Wei QC Li Y He CQ Yang L. Research progress on low-frequency pulsed electromagnetic field therapy for neurological diseases. J Evid Based Med. (2017) 17:373–6,81.

[52] Sherafat MA Heibatollahi M Mongabadi S Moradi F Javan M Ahmadiani A. Electromagnetic field stimulation potentiates endogenous myelin repair by recruiting subventricular neural stem cells in an experimental model of white matter demyelination. J Mol Neurosci. (2012) 48:144–53. doi: 10.1007/s12031-012-9791-8, 22588976

[53] Li JT Dong SQ Qian T Yang WB Chen XJ. Mouse nerve growth factor injection and progression rate in patients with amyotrophic lateral sclerosis: an observational study. Front Neurol. (2022) 13:829569. doi: 10.3389/fneur.2022.829569, 35250834 PMC8891443

[54] Chen SM Chen WL Tai CJ Hsieh SH Lin CK Chen PY . Effects of self-administered acupressure on fatigue following traumatic brain injury: a randomized controlled trial. J Head Trauma Rehabil. (2023) 38:E404–e13. doi: 10.1097/htr.0000000000000861, 36951471

[55] Khodaie F Saeedi R Soleimany G Soleimany MA Kazemi AH Moghadasi AN . Effects of Acupuncture on Cognitive Functions in Patients with Relapsing-Remitting Multiple Sclerosis: A Randomized Controlled Trial. Chin J Integr Med. (2025) 31:928–936. doi: 10.1007/s11655-025-3814-0 39762502

