# Supplementary material for: A network meta-analysis of the performance of acupoint stimulation therapy in improving fatigue, neurological function, and activities of daily living in patients with multiple sclerosis
Source: Front Neurol. 2026 May 26;17:1796876. doi: 10.3389/fneur.2026.1796876 (PMC13247309; doi:10.3389/fneur.2026.1796876)
Supplement: Supplementary file 1 [file Supplementary_file_1.docx]

**Supplementary Materials**

**Supplementary Table 1** Search Strategy

PubMed

| Search number | Query | Sort By | Search Details | Results |
| --- | --- | --- | --- | --- |
| 7 | (("Acupuncture"[Mesh]) OR ("acupoint catgut embedding therapy"[Title/Abstract] OR "acupressure"[Title/Abstract] OR "acupuncture"[Title/Abstract] OR "acupuncture therapy"[Title/Abstract] OR "Acupuncture, Auricular"[Title/Abstract] OR "Acupuncture, Ear"[Title/Abstract] OR "acupuncture, earlobe"[Title/Abstract] OR "acupuncture, electric"[Title/Abstract] OR "Acupunctures, Auricular"[Title/Abstract] OR "Acupunctures, Ear"[Title/Abstract] OR "Auricular Acupuncture"[Title/Abstract] OR "Auricular Acupunctures"[Title/Abstract] OR "auriculoacupuncture"[Title/Abstract] OR "auriculo-acupuncture"[Title/Abstract] OR "auriculotherapy"[Title/Abstract] OR "burnt needle therapy"[Title/Abstract] OR "catgut embedding"[Title/Abstract] OR "catgut implantation"[Title/Abstract] OR "cupping (therapy)"[Title/Abstract] OR "cupping manipulation"[Title/Abstract] OR "Cupping Therapies"[Title/Abstract] OR "Cupping Therapy"[Title/Abstract] OR "Cupping Treatment"[Title/Abstract] OR "Cupping Treatments"[Title/Abstract] OR "Ear Acupuncture"[Title/Abstract] OR "Ear Acupunctures"[Title/Abstract] OR "earlobe acupuncture"[Title/Abstract] OR "electric acupuncture"[Title/Abstract] OR "electrical acupoint stimulation"[Title/Abstract] OR "electrical acupuncture"[Title/Abstract] OR "Electroacupuncture"[Title/Abstract] OR "electro-acupuncture"[Title/Abstract] OR "electrode acupuncture"[Title/Abstract] OR "electronic acupuncture"[Title/Abstract] OR "fire acupuncture"[Title/Abstract] OR "fire cupping"[Title/Abstract] OR "fire needle acupuncture"[Title/Abstract] OR "fire needle therapy"[Title/Abstract] OR "fire needling"[Title/Abstract] OR "flash cupping"[Title/Abstract] OR "heat acupuncture"[Title/Abstract] OR "heat-type acupuncture"[Title/Abstract] OR "massage"[Title/Abstract] OR "Massage Therapies"[Title/Abstract] OR "Massage Therapy"[Title/Abstract] OR "massotherapy"[Title/Abstract] OR "masso-therapy"[Title/Abstract] OR "moving cupping"[Title/Abstract] OR "Moxabustion"[Title/Abstract] OR "moxibustion"[Title/Abstract] OR "Pharmacopuncture"[Title/Abstract] OR "shonishin"[Title/Abstract] OR "sports massage"[Title/Abstract] OR "suction cupping"[Title/Abstract] OR "Therapies, Massage"[Title/Abstract] OR "Therapies, Zone"[Title/Abstract] OR "Therapy, Cupping"[Title/Abstract] OR "Therapy, Massage"[Title/Abstract] OR "Therapy, Zone"[Title/Abstract] OR "thermal acupuncture"[Title/Abstract] OR "thermoacupuncture"[Title/Abstract] OR "thermo-acupuncture"[Title/Abstract] OR "Treatment, Cupping"[Title/Abstract] OR "Tui Na"[Title/Abstract] OR "vacuum cupping"[Title/Abstract] OR "warm acupuncture"[Title/Abstract] OR "warm needle acupuncture"[Title/Abstract] OR "Zone Therapies"[Title/Abstract] OR "Zone Therapy"[Title/Abstract])) AND ((Multiple Sclerosis[MeSH Terms]) OR ("Multiple Sclerosis"[Title/Abstract] OR "chariot disease"[Title/Abstract] OR "Disseminated Sclerosis"[Title/Abstract] OR "insular sclerosis"[Title/Abstract] OR "Multiple Sclerosis, Acute Fulminating"[Title/Abstract] OR "sclerosis multiplex"[Title/Abstract] OR "Sclerosis, Disseminated"[Title/Abstract] OR "sclerosis, insular"[Title/Abstract] OR "Sclerosis, Multiple"[Title/Abstract])) | Publication Date | ("Acupuncture"[MeSH Terms] OR ("acupoint catgut embedding therapy"[Title/Abstract] OR "acupressure"[Title/Abstract] OR "Acupuncture"[Title/Abstract] OR "acupuncture therapy"[Title/Abstract] OR "acupuncture auricular"[Title/Abstract] OR "acupuncture ear"[Title/Abstract] OR "acupuncture electric"[Title/Abstract] OR "Auricular Acupuncture"[Title/Abstract] OR "Auricular Acupunctures"[Title/Abstract] OR "auriculoacupuncture"[Title/Abstract] OR "auriculo-acupuncture"[Title/Abstract] OR "auriculotherapy"[Title/Abstract] OR "burnt needle therapy"[Title/Abstract] OR "catgut embedding"[Title/Abstract] OR "catgut implantation"[Title/Abstract] OR "Cupping Therapy"[Title/Abstract] OR "Cupping Therapies"[Title/Abstract] OR "Cupping Therapy"[Title/Abstract] OR "Cupping Treatment"[Title/Abstract] OR "Cupping Treatments"[Title/Abstract] OR "Ear Acupuncture"[Title/Abstract] OR "electric acupuncture"[Title/Abstract] OR "electrical acupoint stimulation"[Title/Abstract] OR "electrical acupuncture"[Title/Abstract] OR "Electroacupuncture"[Title/Abstract] OR "electro-acupuncture"[Title/Abstract] OR "electronic acupuncture"[Title/Abstract] OR "fire acupuncture"[Title/Abstract] OR "fire cupping"[Title/Abstract] OR "fire needle acupuncture"[Title/Abstract] OR "fire needle therapy"[Title/Abstract] OR "fire needling"[Title/Abstract] OR "flash cupping"[Title/Abstract] OR "heat acupuncture"[Title/Abstract] OR "heat-type acupuncture"[Title/Abstract] OR "massage"[Title/Abstract] OR "Massage Therapies"[Title/Abstract] OR "Massage Therapy"[Title/Abstract] OR "massotherapy"[Title/Abstract] OR "moving cupping"[Title/Abstract] OR "Moxabustion"[Title/Abstract] OR "moxibustion"[Title/Abstract] OR "Pharmacopuncture"[Title/Abstract] OR "sports massage"[Title/Abstract] OR "suction cupping"[Title/Abstract] OR "therapies massage"[Title/Abstract] OR "therapy cupping"[Title/Abstract] OR "therapy massage"[Title/Abstract] OR "therapy zone"[Title/Abstract] OR "thermal acupuncture"[Title/Abstract] OR "thermoacupuncture"[Title/Abstract] OR "thermo-acupuncture"[Title/Abstract] OR "treatment cupping"[Title/Abstract] OR "Tui Na"[Title/Abstract] OR "warm acupuncture"[Title/Abstract] OR "warm needle acupuncture"[Title/Abstract] OR "Zone Therapies"[Title/Abstract] OR "Zone Therapy"[Title/Abstract])) AND ("Multiple Sclerosis"[MeSH Terms] OR ("Multiple Sclerosis"[Title/Abstract] OR "Disseminated Sclerosis"[Title/Abstract] OR "insular sclerosis"[Title/Abstract] OR "multiple sclerosis acute fulminating"[Title/Abstract] OR "sclerosis multiplex"[Title/Abstract] OR "sclerosis disseminated"[Title/Abstract] OR "sclerosis multiple"[Title/Abstract])) | 132 |
| 6 | (Multiple Sclerosis[MeSH Terms]) OR ("Multiple Sclerosis"[Title/Abstract] OR "chariot disease"[Title/Abstract] OR "Disseminated Sclerosis"[Title/Abstract] OR "insular sclerosis"[Title/Abstract] OR "Multiple Sclerosis, Acute Fulminating"[Title/Abstract] OR "sclerosis multiplex"[Title/Abstract] OR "Sclerosis, Disseminated"[Title/Abstract] OR "sclerosis, insular"[Title/Abstract] OR "Sclerosis, Multiple"[Title/Abstract]) | Publication Date | "Multiple Sclerosis"[MeSH Terms] OR "Multiple Sclerosis"[Title/Abstract] OR "Disseminated Sclerosis"[Title/Abstract] OR "insular sclerosis"[Title/Abstract] OR "multiple sclerosis acute fulminating"[Title/Abstract] OR "sclerosis multiplex"[Title/Abstract] OR "sclerosis disseminated"[Title/Abstract] OR "sclerosis multiple"[Title/Abstract] | 110,200 |
| 5 | "Multiple Sclerosis"[Title/Abstract] OR "chariot disease"[Title/Abstract] OR "Disseminated Sclerosis"[Title/Abstract] OR "insular sclerosis"[Title/Abstract] OR "Multiple Sclerosis, Acute Fulminating"[Title/Abstract] OR "sclerosis multiplex"[Title/Abstract] OR "Sclerosis, Disseminated"[Title/Abstract] OR "sclerosis, insular"[Title/Abstract] OR "Sclerosis, Multiple"[Title/Abstract] | Publication Date | "Multiple Sclerosis"[Title/Abstract] OR "Disseminated Sclerosis"[Title/Abstract] OR "insular sclerosis"[Title/Abstract] OR "multiple sclerosis acute fulminating"[Title/Abstract] OR "sclerosis multiplex"[Title/Abstract] OR "sclerosis disseminated"[Title/Abstract] OR "sclerosis multiple"[Title/Abstract] | 102,502 |
| 4 | Multiple Sclerosis[MeSH Terms] | Publication Date | "multiple sclerosis"[MeSH Terms] | 75,681 |
| 3 | ("Acupuncture"[Mesh]) OR ("acupoint catgut embedding therapy"[Title/Abstract] OR "acupressure"[Title/Abstract] OR "acupuncture"[Title/Abstract] OR "acupuncture therapy"[Title/Abstract] OR "Acupuncture, Auricular"[Title/Abstract] OR "Acupuncture, Ear"[Title/Abstract] OR "acupuncture, earlobe"[Title/Abstract] OR "acupuncture, electric"[Title/Abstract] OR "Acupunctures, Auricular"[Title/Abstract] OR "Acupunctures, Ear"[Title/Abstract] OR "Auricular Acupuncture"[Title/Abstract] OR "Auricular Acupunctures"[Title/Abstract] OR "auriculoacupuncture"[Title/Abstract] OR "auriculo-acupuncture"[Title/Abstract] OR "auriculotherapy"[Title/Abstract] OR "burnt needle therapy"[Title/Abstract] OR "catgut embedding"[Title/Abstract] OR "catgut implantation"[Title/Abstract] OR "cupping (therapy)"[Title/Abstract] OR "cupping manipulation"[Title/Abstract] OR "Cupping Therapies"[Title/Abstract] OR "Cupping Therapy"[Title/Abstract] OR "Cupping Treatment"[Title/Abstract] OR "Cupping Treatments"[Title/Abstract] OR "Ear Acupuncture"[Title/Abstract] OR "Ear Acupunctures"[Title/Abstract] OR "earlobe acupuncture"[Title/Abstract] OR "electric acupuncture"[Title/Abstract] OR "electrical acupoint stimulation"[Title/Abstract] OR "electrical acupuncture"[Title/Abstract] OR "Electroacupuncture"[Title/Abstract] OR "electro-acupuncture"[Title/Abstract] OR "electrode acupuncture"[Title/Abstract] OR "electronic acupuncture"[Title/Abstract] OR "fire acupuncture"[Title/Abstract] OR "fire cupping"[Title/Abstract] OR "fire needle acupuncture"[Title/Abstract] OR "fire needle therapy"[Title/Abstract] OR "fire needling"[Title/Abstract] OR "flash cupping"[Title/Abstract] OR "heat acupuncture"[Title/Abstract] OR "heat-type acupuncture"[Title/Abstract] OR "massage"[Title/Abstract] OR "Massage Therapies"[Title/Abstract] OR "Massage Therapy"[Title/Abstract] OR "massotherapy"[Title/Abstract] OR "masso-therapy"[Title/Abstract] OR "moving cupping"[Title/Abstract] OR "Moxabustion"[Title/Abstract] OR "moxibustion"[Title/Abstract] OR "Pharmacopuncture"[Title/Abstract] OR "shonishin"[Title/Abstract] OR "sports massage"[Title/Abstract] OR "suction cupping"[Title/Abstract] OR "Therapies, Massage"[Title/Abstract] OR "Therapies, Zone"[Title/Abstract] OR "Therapy, Cupping"[Title/Abstract] OR "Therapy, Massage"[Title/Abstract] OR "Therapy, Zone"[Title/Abstract] OR "thermal acupuncture"[Title/Abstract] OR "thermoacupuncture"[Title/Abstract] OR "thermo-acupuncture"[Title/Abstract] OR "Treatment, Cupping"[Title/Abstract] OR "Tui Na"[Title/Abstract] OR "vacuum cupping"[Title/Abstract] OR "warm acupuncture"[Title/Abstract] OR "warm needle acupuncture"[Title/Abstract] OR "Zone Therapies"[Title/Abstract] OR "Zone Therapy"[Title/Abstract]) | Publication Date | "Acupuncture"[MeSH Terms] OR "acupoint catgut embedding therapy"[Title/Abstract] OR "acupressure"[Title/Abstract] OR "Acupuncture"[Title/Abstract] OR "acupuncture therapy"[Title/Abstract] OR "acupuncture auricular"[Title/Abstract] OR "acupuncture ear"[Title/Abstract] OR "acupuncture electric"[Title/Abstract] OR "Auricular Acupuncture"[Title/Abstract] OR "Auricular Acupunctures"[Title/Abstract] OR "auriculoacupuncture"[Title/Abstract] OR "auriculo-acupuncture"[Title/Abstract] OR "auriculotherapy"[Title/Abstract] OR "burnt needle therapy"[Title/Abstract] OR "catgut embedding"[Title/Abstract] OR "catgut implantation"[Title/Abstract] OR "Cupping Therapy"[Title/Abstract] OR "Cupping Therapies"[Title/Abstract] OR "Cupping Therapy"[Title/Abstract] OR "Cupping Treatment"[Title/Abstract] OR "Cupping Treatments"[Title/Abstract] OR "Ear Acupuncture"[Title/Abstract] OR "electric acupuncture"[Title/Abstract] OR "electrical acupoint stimulation"[Title/Abstract] OR "electrical acupuncture"[Title/Abstract] OR "Electroacupuncture"[Title/Abstract] OR "electro-acupuncture"[Title/Abstract] OR "electronic acupuncture"[Title/Abstract] OR "fire acupuncture"[Title/Abstract] OR "fire cupping"[Title/Abstract] OR "fire needle acupuncture"[Title/Abstract] OR "fire needle therapy"[Title/Abstract] OR "fire needling"[Title/Abstract] OR "flash cupping"[Title/Abstract] OR "heat acupuncture"[Title/Abstract] OR "heat-type acupuncture"[Title/Abstract] OR "massage"[Title/Abstract] OR "Massage Therapies"[Title/Abstract] OR "Massage Therapy"[Title/Abstract] OR "massotherapy"[Title/Abstract] OR "moving cupping"[Title/Abstract] OR "Moxabustion"[Title/Abstract] OR "moxibustion"[Title/Abstract] OR "Pharmacopuncture"[Title/Abstract] OR "sports massage"[Title/Abstract] OR "suction cupping"[Title/Abstract] OR "therapies massage"[Title/Abstract] OR "therapy cupping"[Title/Abstract] OR "therapy massage"[Title/Abstract] OR "therapy zone"[Title/Abstract] OR "thermal acupuncture"[Title/Abstract] OR "thermoacupuncture"[Title/Abstract] OR "thermo-acupuncture"[Title/Abstract] OR "treatment cupping"[Title/Abstract] OR "Tui Na"[Title/Abstract] OR "warm acupuncture"[Title/Abstract] OR "warm needle acupuncture"[Title/Abstract] OR "Zone Therapies"[Title/Abstract] OR "Zone Therapy"[Title/Abstract] | 51,683 |
| 2 | "acupoint catgut embedding therapy"[Title/Abstract] OR "acupressure"[Title/Abstract] OR "acupuncture"[Title/Abstract] OR "acupuncture therapy"[Title/Abstract] OR "Acupuncture, Auricular"[Title/Abstract] OR "Acupuncture, Ear"[Title/Abstract] OR "acupuncture, earlobe"[Title/Abstract] OR "acupuncture, electric"[Title/Abstract] OR "Acupunctures, Auricular"[Title/Abstract] OR "Acupunctures, Ear"[Title/Abstract] OR "Auricular Acupuncture"[Title/Abstract] OR "Auricular Acupunctures"[Title/Abstract] OR "auriculoacupuncture"[Title/Abstract] OR "auriculo-acupuncture"[Title/Abstract] OR "auriculotherapy"[Title/Abstract] OR "burnt needle therapy"[Title/Abstract] OR "catgut embedding"[Title/Abstract] OR "catgut implantation"[Title/Abstract] OR "cupping (therapy)"[Title/Abstract] OR "cupping manipulation"[Title/Abstract] OR "Cupping Therapies"[Title/Abstract] OR "Cupping Therapy"[Title/Abstract] OR "Cupping Treatment"[Title/Abstract] OR "Cupping Treatments"[Title/Abstract] OR "Ear Acupuncture"[Title/Abstract] OR "Ear Acupunctures"[Title/Abstract] OR "earlobe acupuncture"[Title/Abstract] OR "electric acupuncture"[Title/Abstract] OR "electrical acupoint stimulation"[Title/Abstract] OR "electrical acupuncture"[Title/Abstract] OR "Electroacupuncture"[Title/Abstract] OR "electro-acupuncture"[Title/Abstract] OR "electrode acupuncture"[Title/Abstract] OR "electronic acupuncture"[Title/Abstract] OR "fire acupuncture"[Title/Abstract] OR "fire cupping"[Title/Abstract] OR "fire needle acupuncture"[Title/Abstract] OR "fire needle therapy"[Title/Abstract] OR "fire needling"[Title/Abstract] OR "flash cupping"[Title/Abstract] OR "heat acupuncture"[Title/Abstract] OR "heat-type acupuncture"[Title/Abstract] OR "massage"[Title/Abstract] OR "Massage Therapies"[Title/Abstract] OR "Massage Therapy"[Title/Abstract] OR "massotherapy"[Title/Abstract] OR "masso-therapy"[Title/Abstract] OR "moving cupping"[Title/Abstract] OR "Moxabustion"[Title/Abstract] OR "moxibustion"[Title/Abstract] OR "Pharmacopuncture"[Title/Abstract] OR "shonishin"[Title/Abstract] OR "sports massage"[Title/Abstract] OR "suction cupping"[Title/Abstract] OR "Therapies, Massage"[Title/Abstract] OR "Therapies, Zone"[Title/Abstract] OR "Therapy, Cupping"[Title/Abstract] OR "Therapy, Massage"[Title/Abstract] OR "Therapy, Zone"[Title/Abstract] OR "thermal acupuncture"[Title/Abstract] OR "thermoacupuncture"[Title/Abstract] OR "thermo-acupuncture"[Title/Abstract] OR "Treatment, Cupping"[Title/Abstract] OR "Tui Na"[Title/Abstract] OR "vacuum cupping"[Title/Abstract] OR "warm acupuncture"[Title/Abstract] OR "warm needle acupuncture"[Title/Abstract] OR "Zone Therapies"[Title/Abstract] OR "Zone Therapy"[Title/Abstract] | Publication Date | "acupoint catgut embedding therapy"[Title/Abstract] OR "acupressure"[Title/Abstract] OR "acupuncture"[Title/Abstract] OR "acupuncture therapy"[Title/Abstract] OR "acupuncture auricular"[Title/Abstract] OR "acupuncture ear"[Title/Abstract] OR "acupuncture electric"[Title/Abstract] OR "Auricular Acupuncture"[Title/Abstract] OR "Auricular Acupunctures"[Title/Abstract] OR "auriculoacupuncture"[Title/Abstract] OR "auriculo-acupuncture"[Title/Abstract] OR "auriculotherapy"[Title/Abstract] OR "burnt needle therapy"[Title/Abstract] OR "catgut embedding"[Title/Abstract] OR "catgut implantation"[Title/Abstract] OR "Cupping Therapy"[Title/Abstract] OR "Cupping Therapies"[Title/Abstract] OR "Cupping Therapy"[Title/Abstract] OR "Cupping Treatment"[Title/Abstract] OR "Cupping Treatments"[Title/Abstract] OR "Ear Acupuncture"[Title/Abstract] OR "electric acupuncture"[Title/Abstract] OR "electrical acupoint stimulation"[Title/Abstract] OR "electrical acupuncture"[Title/Abstract] OR "Electroacupuncture"[Title/Abstract] OR "electro-acupuncture"[Title/Abstract] OR "electronic acupuncture"[Title/Abstract] OR "fire acupuncture"[Title/Abstract] OR "fire cupping"[Title/Abstract] OR "fire needle acupuncture"[Title/Abstract] OR "fire needle therapy"[Title/Abstract] OR "fire needling"[Title/Abstract] OR "flash cupping"[Title/Abstract] OR "heat acupuncture"[Title/Abstract] OR "heat-type acupuncture"[Title/Abstract] OR "massage"[Title/Abstract] OR "Massage Therapies"[Title/Abstract] OR "Massage Therapy"[Title/Abstract] OR "massotherapy"[Title/Abstract] OR "moving cupping"[Title/Abstract] OR "Moxabustion"[Title/Abstract] OR "moxibustion"[Title/Abstract] OR "Pharmacopuncture"[Title/Abstract] OR "sports massage"[Title/Abstract] OR "suction cupping"[Title/Abstract] OR "therapies massage"[Title/Abstract] OR "therapy cupping"[Title/Abstract] OR "therapy massage"[Title/Abstract] OR "therapy zone"[Title/Abstract] OR "thermal acupuncture"[Title/Abstract] OR "thermoacupuncture"[Title/Abstract] OR "thermo-acupuncture"[Title/Abstract] OR "treatment cupping"[Title/Abstract] OR "Tui Na"[Title/Abstract] OR "warm acupuncture"[Title/Abstract] OR "warm needle acupuncture"[Title/Abstract] OR "Zone Therapies"[Title/Abstract] OR "Zone Therapy"[Title/Abstract] | 51,466 |
| 1 | "Acupuncture"[Mesh] | Most Recent | "Acupuncture"[MeSH Terms] | 2,116 |

**Embase**

| No. | Query | Results |
| --- | --- | --- |
| #7 | #3 AND #6 | 422 |
| #6 | #4 OR #5 | 196902 |
| #5 | 'multiple sclerosis':ab,ti OR 'chariot disease':ab,ti OR 'disseminated sclerosis':ab,ti OR 'insular sclerosis':ab,ti OR 'multiple sclerosis, acute fulminating':ab,ti OR 'sclerosis multiplex':ab,ti OR 'sclerosis, disseminated':ab,ti OR 'sclerosis, insular':ab,ti OR 'sclerosis, multiple':ab,ti | 161095 |
| #4 | 'multiple sclerosis'/exp | 181747 |
| #3 | #1 OR #2 | 88385 |
| #2 | ('acupoint catgut embedding therapy':ab,ti OR 'acupressure':ab,ti OR 'acupuncture':ab,ti OR 'acupuncture therapy':ab,ti OR 'acupuncture, auricular':ab,ti OR 'acupuncture, ear':ab,ti OR 'acupuncture, earlobe':ab,ti OR 'acupuncture, electric':ab,ti OR 'acupunctures, auricular':ab,ti OR 'acupunctures, ear':ab,ti OR 'auricular acupuncture':ab,ti OR 'auricular acupunctures':ab,ti OR 'auriculoacupuncture':ab,ti OR 'auriculo-acupuncture':ab,ti OR 'auriculotherapy':ab,ti OR 'burnt needle therapy':ab,ti OR 'catgut embedding':ab,ti OR 'catgut implantation':ab,ti OR cupping:ab,ti) AND therapy:ab,ti OR 'cupping manipulation':ab,ti OR 'cupping therapies':ab,ti OR 'cupping therapy':ab,ti OR 'cupping treatment':ab,ti OR 'cupping treatments':ab,ti OR 'ear acupuncture':ab,ti OR 'ear acupunctures':ab,ti OR 'earlobe acupuncture':ab,ti OR 'electric acupuncture':ab,ti OR 'electrical acupoint stimulation':ab,ti OR 'electrical acupuncture':ab,ti OR 'electroacupuncture':ab,ti OR 'electro-acupuncture':ab,ti OR 'electrode acupuncture':ab,ti OR 'electronic acupuncture':ab,ti OR 'fire acupuncture':ab,ti OR 'fire cupping':ab,ti OR 'fire needle acupuncture':ab,ti OR 'fire needle therapy':ab,ti OR 'fire needling':ab,ti OR 'flash cupping':ab,ti OR 'heat acupuncture':ab,ti OR 'heat-type acupuncture':ab,ti OR 'massage':ab,ti OR 'massage therapies':ab,ti OR 'massage therapy':ab,ti OR 'massotherapy':ab,ti OR 'masso-therapy':ab,ti OR 'moving cupping':ab,ti OR 'moxabustion':ab,ti OR 'moxibustion':ab,ti OR 'pharmacopuncture':ab,ti OR 'shonishin':ab,ti OR 'sports massage':ab,ti OR 'suction cupping':ab,ti OR 'therapies, massage':ab,ti OR 'therapies, zone':ab,ti OR 'therapy, cupping':ab,ti OR 'therapy, massage':ab,ti OR 'therapy, zone':ab,ti OR 'thermal acupuncture':ab,ti OR 'thermoacupuncture':ab,ti OR 'thermo-acupuncture':ab,ti OR 'treatment, cupping':ab,ti OR 'tui na':ab,ti OR 'vacuum cupping':ab,ti OR 'warm acupuncture':ab,ti OR 'warm needle acupuncture':ab,ti OR 'zone therapies':ab,ti OR 'zone therapy':ab,ti | 46452 |
| #1 | 'acupuncture'/exp | 67076 |

Cohrane Library

| No. | Query | Results |
| --- | --- | --- |
| #7 | #3 AND #6 | 128 |
| #6 | #4 OR #5 | 34698 |
| #5 | ("acupoint catgut embedding therapy" OR "acupressure" OR "acupuncture" OR "acupuncture therapy" OR "Acupuncture, Auricular" OR "Acupuncture, Ear" OR "acupuncture, earlobe" OR "acupuncture, electric" OR "Acupunctures, Auricular" OR "Acupunctures, Ear" OR "Auricular Acupuncture" OR "Auricular Acupunctures" OR "auriculoacupuncture" OR "auriculo-acupuncture" OR "auriculotherapy" OR "burnt needle therapy" OR "catgut embedding" OR "catgut implantation" OR "cupping (therapy)" OR "cupping manipulation" OR "Cupping Therapies" OR "Cupping Therapy" OR "Cupping Treatment" OR "Cupping Treatments" OR "Ear Acupuncture" OR "Ear Acupunctures" OR "earlobe acupuncture" OR "electric acupuncture" OR "electrical acupoint stimulation" OR "electrical acupuncture" OR "Electroacupuncture" OR "electro-acupuncture" OR "electrode acupuncture" OR "electronic acupuncture" OR "fire acupuncture" OR "fire cupping" OR "fire needle acupuncture" OR "fire needle therapy" OR "fire needling" OR "flash cupping" OR "heat acupuncture" OR "heat-type acupuncture" OR "massage" OR "Massage Therapies" OR "Massage Therapy" OR "massotherapy" OR "masso-therapy" OR "moving cupping" OR "Moxabustion" OR "moxibustion" OR "Pharmacopuncture" OR "shonishin" OR "sports massage" OR "suction cupping" OR "Therapies, Massage" OR "Therapies, Zone" OR "Therapy, Cupping" OR "Therapy, Massage" OR "Therapy, Zone" OR "thermal acupuncture" OR "thermoacupuncture" OR "thermo-acupuncture" OR "Treatment, Cupping" OR "Tui Na" OR "vacuum cupping" OR "warm acupuncture" OR "warm needle acupuncture" OR "Zone Therapies" OR "Zone Therapy"):ti,ab,kw | 34698 |
| #4 | MeSH descriptor: [Acupuncture] explode all trees | 216 |
| #3 | #1 OR #2 | 13362 |
| #2 | ("Multiple Sclerosis" OR "chariot disease" OR "Disseminated Sclerosis" OR "insular sclerosis" OR "Multiple Sclerosis, Acute Fulminating" OR "sclerosis multiplex" OR "Sclerosis, Disseminated" OR "sclerosis, insular" OR "Sclerosis, Multiple"):ti,ab,kw | 13362 |
| #1 | MeSH descriptor: [Multiple Sclerosis] explode all trees | 5424 |

Web of science

| No. | Query | Results |
| --- | --- | --- |
| 1 | "Multiple Sclerosis" OR "chariot disease" OR "Disseminated Sclerosis" OR "insular sclerosis" OR "Multiple Sclerosis, Acute Fulminating" OR "sclerosis multiplex" OR "Sclerosis, Disseminated" OR "sclerosis, insular" OR "Sclerosis, Multiple" (Topic) and "acupoint catgut embedding therapy" OR "acupressure" OR "acupuncture" OR "acupuncture therapy" OR "Acupuncture, Auricular" OR "Acupuncture, Ear" OR "acupuncture, earlobe" OR "acupuncture, electric" OR "Acupunctures, Auricular" OR "Acupunctures, Ear" OR "Auricular Acupuncture" OR "Auricular Acupunctures" OR "auriculoacupuncture" OR "auriculo-acupuncture" OR "auriculotherapy" OR "burnt needle therapy" OR "catgut embedding" OR "catgut implantation" OR "cupping (therapy)" OR "cupping manipulation" OR "Cupping Therapies" OR "Cupping Therapy" OR "Cupping Treatment" OR "Cupping Treatments" OR "Ear Acupuncture" OR "Ear Acupunctures" OR "earlobe acupuncture" OR "electric acupuncture" OR "electrical acupoint stimulation" OR "electrical acupuncture" OR "Electroacupuncture" OR "electro-acupuncture" OR "electrode acupuncture" OR "electronic acupuncture" OR "fire acupuncture" OR "fire cupping" OR "fire needle acupuncture" OR "fire needle therapy" OR "fire needling" OR "flash cupping" OR "heat acupuncture" OR "heat-type acupuncture" OR "massage" OR "Massage Therapies" OR "Massage Therapy" OR "massotherapy" OR "masso-therapy" OR "moving cupping" OR "Moxabustion" OR "moxibustion" OR "Pharmacopuncture" OR "shonishin" OR "sports massage" OR "suction cupping" OR "Therapies, Massage" OR "Therapies, Zone" OR "Therapy, Cupping" OR "Therapy, Massage" OR "Therapy, Zone" OR "thermal acupuncture" OR "thermoacupuncture" OR "thermo-acupuncture" OR "Treatment, Cupping" OR "Tui Na" OR "vacuum cupping" OR "warm acupuncture" OR "warm needle acupuncture" OR "Zone Therapies" OR "Zone Therapy" (Topic) and Preprint Citation Index (Exclude – Database) | 278 |

**VIP**

| No. | Query | Results |
| --- | --- | --- |
| 1 | [(题名或关键词=多发性硬化 AND ((((((((((((((((((((((((((((((题名或关键词=针刺 OR 题名或关键词=针灸) OR 题名或关键词=针法) OR 题名或关键词=艾灸) OR 题名或关键词=灸法) OR 题名或关键词=按摩) OR 题名或关键词=按摩疗法) OR 题名或关键词=推拿治疗) OR 题名或关键词=推拿疗法) OR 题名或关键词=穴位按压) OR 题名或关键词=推拿) OR 题名或关键词=拔罐疗法) OR 题名或关键词=拔罐) OR 题名或关键词=拔火罐) OR 题名或关键词=拔罐子) OR 题名或关键词=刮痧) OR 题名或关键词=电针) OR 题名或关键词=温针灸) OR 题名或关键词=穴位注射) OR 题名或关键词=穴位贴敷) OR 题名或关键词=穴位敷贴) OR 题名或关键词=穴位埋线) OR 题名或关键词=穴位放血) OR 题名或关键词=穴位磁疗) OR 题名或关键词=穴位离子导入) OR 题名或关键词=火针) OR 题名或关键词=燔针) OR 题名或关键词=耳穴) OR 题名或关键词=耳压) OR 题名或关键词=耳埋) OR 题名或关键词=耳针))](https://qikan.cqvip.com/Qikan/search/index?LngMySearHistoryIdGuid=a55dd0e6-5401-4236-9c4a-4fad1f704a8b&from=Qikan_Article_History) | 83 |

CNKI

| No. | Query | Results |
| --- | --- | --- |
| 1 | （篇关摘：多发性硬化(精确)）AND（篇关摘：针刺 + 针灸 + 针法 + 艾灸 + 灸法 + 按摩 + 按摩疗法 + 推拿治疗 + 推拿疗法 + 穴位按压 + 推拿 + 拔罐疗法 + 拔罐 + 拔火罐 + 拔罐子 + 刮痧 + 电针 + 温针灸 + 穴位注射 + 穴位贴敷 + 穴位敷贴 + 穴位埋线 + 穴位放血 + 穴位磁疗 + 穴位离子导入 + 火针 + 燔针 + 耳穴 + 耳压 + 耳埋 + 耳针(精确)） | 182 |

**Wanfang Data**

| No. | Query | Results |
| --- | --- | --- |
| 1 | 题名或关键词:(多发性硬化) and 题名或关键词:(针刺 OR 针灸 OR 针法 OR 艾灸 OR 灸法 OR 按摩 OR 按摩疗法 OR 推拿治疗 OR 推拿疗法 OR 穴位按压 OR 推拿 OR 拔罐疗法 OR 拔罐 OR 拔火罐 OR 拔罐子 OR 刮痧 OR 电针 OR 温针灸 OR 穴位注射 OR 穴位贴敷 OR 穴位敷贴 OR 穴位埋线 OR 穴位放血 OR 穴位磁疗 OR 穴位离子导入 OR 火针 OR 燔针 OR 耳穴 OR 耳压 OR 耳埋 OR 耳针 OR 温针) | 253 |

**SinoMed**

| 序号 | 检索表达式 | 命中文献数 |
| --- | --- | --- |
| 1) | "多发性硬化"[不加权:扩展] | 79315 |
| 2) | "针刺"[不加权:扩展] | 1082 |
| 3) | "多发性硬化"[常用字段:智能] | 116783 |
| 4) | ( "针刺"[常用字段:智能] OR "针灸"[常用字段:智能] OR "针法"[常用字段:智能] OR "艾灸"[常用字段:智能] OR "灸法"[常用字段:智能] OR "按摩"[常用字段:智能] OR "按摩疗法"[常用字段:智能] OR "推拿治疗"[常用字段:智能] OR "推拿疗法"[常用字段:智能] OR "穴位按压"[常用字段:智能] OR "推拿"[常用字段:智能] OR "拔罐疗法"[常用字段:智能] OR "拔罐"[常用字段:智能] OR "拔火罐"[常用字段:智能] OR "拔罐子"[常用字段:智能] OR "刮痧"[常用字段:智能] OR "电针"[常用字段:智能] OR "温针灸"[常用字段:智能] OR "穴位注射"[常用字段:智能] OR "穴位贴敷"[常用字段:智能] OR "穴位敷贴"[常用字段:智能] OR "穴位埋线"[常用字段:智能] OR "穴位放血"[常用字段:智能] OR "穴位磁疗"[常用字段:智能] OR "穴位离子导入"[常用字段:智能] OR "火针"[常用字段:智能] OR "燔针"[常用字段:智能] OR "耳穴"[常用字段:智能] OR "耳压"[常用字段:智能] OR "耳埋"[常用字段:智能] OR "耳针"[常用字段:智能] OR "温针"[常用字段:智能]) | 480229 |
| 5) | (("多发性硬化"[不加权:扩展]) OR ("多发性硬化"[常用字段:智能])) | 116783 |
| 6) | (("针刺"[不加权:扩展]) OR ((( "针刺"[常用字段:智能] OR "针灸"[常用字段:智能] OR "针法"[常用字段:智能] OR "艾灸"[常用字段:智能] OR "灸法"[常用字段:智能] OR "按摩"[常用字段:智能] OR "按摩疗法"[常用字段:智能] OR "推拿治疗"[常用字段:智能] OR "推拿疗法"[常用字段:智能] OR "穴位按压"[常用字段:智能] OR "推拿"[常用字段:智能] OR "拔罐疗法"[常用字段:智能] OR "拔罐"[常用字段:智能] OR "拔火罐"[常用字段:智能] OR "拔罐子"[常用字段:智能] OR "刮痧"[常用字段:智能] OR "电针"[常用字段:智能] OR "温针灸"[常用字段:智能] OR "穴位注射"[常用字段:智能] OR "穴位贴敷"[常用字段:智能] OR "穴位敷贴"[常用字段:智能] OR "穴位埋线"[常用字段:智能] OR "穴位放血"[常用字段:智能] OR "穴位磁疗"[常用字段:智能] OR "穴位离子导入"[常用字段:智能] OR "火针"[常用字段:智能] OR "燔针"[常用字段:智能] OR "耳穴"[常用字段:智能] OR "耳压"[常用字段:智能] OR "耳埋"[常用字段:智能] OR "耳针"[常用字段:智能] OR "温针"[常用字段:智能])) OR ("针刺"[不加权:扩展]))) | 480229 |
| 7) | ((("针刺"[不加权:扩展]) OR ((( "针刺"[常用字段:智能] OR "针灸"[常用字段:智能] OR "针法"[常用字段:智能] OR "艾灸"[常用字段:智能] OR "灸法"[常用字段:智能] OR "按摩"[常用字段:智能] OR "按摩疗法"[常用字段:智能] OR "推拿治疗"[常用字段:智能] OR "推拿疗法"[常用字段:智能] OR "穴位按压"[常用字段:智能] OR "推拿"[常用字段:智能] OR "拔罐疗法"[常用字段:智能] OR "拔罐"[常用字段:智能] OR "拔火罐"[常用字段:智能] OR "拔罐子"[常用字段:智能] OR "刮痧"[常用字段:智能] OR "电针"[常用字段:智能] OR "温针灸"[常用字段:智能] OR "穴位注射"[常用字段:智能] OR "穴位贴敷"[常用字段:智能] OR "穴位敷贴"[常用字段:智能] OR "穴位埋线"[常用字段:智能] OR "穴位放血"[常用字段:智能] OR "穴位磁疗"[常用字段:智能] OR "穴位离子导入"[常用字段:智能] OR "火针"[常用字段:智能] OR "燔针"[常用字段:智能] OR "耳穴"[常用字段:智能] OR "耳压"[常用字段:智能] OR "耳埋"[常用字段:智能] OR "耳针"[常用字段:智能] OR "温针"[常用字段:智能])) OR ("针刺"[不加权:扩展])))) AND ((("多发性硬化"[不加权:扩展]) OR ("多发性硬化"[常用字段:智能]))) | 303 |

**Supplementary Table 2** Specific definitions of standard treatment

| Study ID | Standard treatment |
| --- | --- |
| Xu 2011 | Methylprednisolone, prednisone |
| Hu 2010 | Methylprednisolone, prednisone |
| Cui 2013 | Baclofen tablets |
| Wang 2017 | Basic symptomatic and supportive care; details not specified |
| Li 2016 | Basic symptomatic and supportive care; details not specified |
| Li 2013 | Methylprednisolone |
| Zhou 2017 | Methylprednisolone, prednisone acetate tablets |
| Ding 2013 | Basic symptomatic and supportive care; details not specified |
| Li 2020 | Mecobalamin injection, methylprednisolone sodium succinate for injection |
| Yang 2014 | Treatments such as corticosteroids, interferon-β, azathioprine, immunoglobulin, and plasma exchange, as well as symptomatic care and daily training |
| Zheng 2013 | Prednisone/methylprednisolone, immunoglobulin, methotrexate, cyclophosphamide, cyclosporine A, and interferon-β |
| Luo 2015 | Basic symptomatic and supportive care; details not specified |
| Wang 2016 | Fingolimod, amitriptylin |
| Wu 2015 | Methylprednisolone sodium succinate, prednisone acetate |
| Ran 2018 | /Methylprednisolone sodium succinate, prednisone |
| Cabanillas 2012 | Basic symptomatic and supportive care; details not specified |
| Yeni 2022 | Basic symptomatic and supportive care; details not specified |
| Rahimi 2020 | Basic symptomatic and supportive care; details not specified |
| Khodaie 2024 | Rituximab, ocrelizumab, natalizumab, fingolimod, interferon β-1a, glatiramer acetate, dimethyl fumarate, and mycophenolate mofetil |
| Khodaie 2023 | Amantadine |
| Bastani 2015 | Basic symptomatic and supportive care; details not specified |
| Donnellan 2008 | Basic symptomatic and supportive care; details not specified |
| Sungur 2023 | Basic symptomatic and supportive care; details not specified |

**Supplementary Table 3** Specific definitions of response rate

| Study ID | Assessment tool | Grading criteria for treatment efficacy | Calculation method for reponse rate |
| --- | --- | --- | --- |
| Xu 2011 | EDSS | Calculated as [(pre-treatment score – post-treatment score) / pre-treatment score] × 100%, expressed as a percentage. Complete remission: >85%; marked response: 50%–85%; effective: 20%–49%; ineffective: <20%. | Overall response rate = [(complete remission + marked response + effective) / total number of cases] × 100%. |
| Li 2013 | EDSS | Calculated as [(pre-treatment score – post-treatment score) / pre-treatment score] × 100%, expressed as a percentage. Marked response: 100%–60%; effective: 60%–30%; ineffective: <30% | Overall response rate = [(marked response + effective) / total number of cases] × 100%. |
| Zhou 2017 | EDSS | Calculated as [(pre-treatment score – post-treatment score) / pre-treatment score] × 100%, expressed as a percentage. Complete remission: >85%; marked response: 50%–85%; effective: 20%–49%; ineffective: <20%. | Overall response rate = [(complete remission + marked response + effective) / total number of cases] × 100%. |
| Li 2020 | EDSS | The specific definitions of “marked response,” “effective,” and “ineffective” are not specified. | Overall response rate = [(marked response + effective) / total number of cases] × 100%. |
| Yang 2014 | Clinical assessment | Marked response: Symptoms and signs (e.g., fatigue) have basically disappeared; limitations in activity are not significant; muscle strength is nearly normal. Effective: Symptoms (e.g., fatigue) have been moderately alleviated but have not completely disappeared; activity and muscle strength are mildly limited. Ineffective: Symptoms (e.g., fatigue) show no significant alleviation; muscle atrophy gradually worsens; muscle strength is significantly limited. | Overall response rate = [(marked response + effective) / total number of cases] × 100%. |
| Zheng 2013 | Clinical assessment | Rapid response: Clinical symptoms rapidly recover to near-normal levels within 2–3 months; Marked response: Major clinical symptoms recover to near-normal levels within 4–8 months; Effective: Major clinical symptoms recover to near-normal levels within 8–10 months; Ineffective: No significant alleviation is observed in major clinical symptoms after 12 months or more. | Overall response rate = [(rapid response + marked response + effective) / total number of cases] × 100%. |
| Wu 2015 | Clinical assessment | Marked response: Major clinical symptoms and signs show significantly alleviation; the patient’s neurological function is substantially restored; and the patient’s quality of life is significantly enhanced. Effective: Major clinical symptoms and signs have been alleviated to some extent; the patient’s neurological function is partially restored; and the patient’s quality of life is improved to some extent. Ineffective: Major clinical symptoms and signs have not been alleviated; the patient’s neurological function has not been effectively restored; and the patient’s quality of life has not shown significant improvement or has even worsened. | Overall response rate = [(marked response + effective) / total number of cases] × 100%. |
| Ran 2018 | EDSS | Calculated as [(pre-treatment score – post-treatment score) / pre-treatment score] × 100%, expressed as a percentage. Complete remission: >85%; marked response: 50%–85%; effective: 20%–49%; ineffective: <20%. | Overall response rate = [(complete remission + marked response + effective) / total number of cases] × 100%. |

**Supplementary Table 4** Table of Acupoint Application Frequency

| **Acupoint Code** | **Acupoint Name** | **Frequency** |  | **Acupoint Code** | **Acupoint Name** | **Frequency** |
| --- | --- | --- | --- | --- | --- | --- |
| SP 6 | Sanyinjiao | 17 |  | GV 10 | Lingtai | 2 |
| ST 36 | Zusanli | 17 |  | GV 7 | Zhongshu | 2 |
| GV 20 | Baihui | 10 |  | LU5 | Chize | 2 |
| LI 4 | Hegu | 10 |  | MS7 | Jiaogan | 2 |
| GB 20 | Fengchi | 7 |  | SI 3 | Houxi | 2 |
| PC 6 | Neiguan | 7 |  | SP 10 | Xuehai | 2 |
| LI 11 | Quchi | 6 |  | ST 25 | Tianshu | 2 |
| LR 3 | Taichong | 6 |  | ST 31 | Biguan | 2 |
| EX-B2 | Jiaji | 5 |  | TE 5 | Waiguan | 2 |
| GB 34 | Yanglingquan | 5 |  | BL 22 | Sanjiaoshu | 1 |
| BL 18 | Ganshu | 4 |  | BL 40 | Weizhong | 1 |
| BL 23 | Shenshu | 4 |  | BL 62 | Shenmai | 1 |
| CV 6 | Qihai | 3 |  | BL15 | Xinshu | 1 |
| EX-HN 3 | Yintang | 3 |  | BL16 | Dushu | 1 |
| GV 11 | Shendao | 3 |  | BL17 | Geshu | 1 |
| GV 12 | Shenzhu | 3 |  | CV 10 | Xiawan | 1 |
| GV 13 | Taodao | 3 |  | EX-HN1 | Sishencong | 1 |
| GV 14 | Dazhui | 3 |  | GB 30 | Huantiao | 1 |
| GV 3 | Yaoyangguan | 3 |  | GB 31 | Fengshi | 1 |
| GV 4 | Mingmen | 3 |  | GB 39 | Xuanzhong | 1 |
| GV 5 | Xuanshu | 3 |  | GB 41 | Zulinqi | 1 |
| GV 6 | Jizhong | 3 |  | GV 1 | Changqiang | 1 |
| GV 8 | Jinsuo | 3 |  | GV 15 | Yamen | 1 |
| GV 9 | Zhiyang | 3 |  | GV 16 | Fengfu | 1 |
| HT 7 | Shenmen | 3 |  | GV 24 | Shenting | 1 |
| KI 3 | Taixi | 3 |  | GV 26 | Shuigou | 1 |
| BL 10 | Tianzhu | 2 |  | HT 1 | Jiquan | 1 |
| BL 19 | Danshu | 2 |  | KI 1 | Yongquan | 1 |
| BL 20 | Pishu | 2 |  | LI 15 | Jianyu | 1 |
| BL 21 | Weishu | 2 |  | MS6 | Wan | 1 |
| BL 60 | Kunlun | 2 |  | SI 3 | Houxi | 1 |
| CV 12 | Zhongwan | 2 |  | ST 32 | Futu | 1 |
| CV 13 | Shangwan | 2 |  | ST 35 | Dubi | 1 |
| CV4 | Guanyuan | 2 |  | ST 40 | Fenglong | 1 |
| GB 15 | Toulinqi | 2 |  | ST 40 | Fenglong | 1 |
| GB 8 | Shuaigu | 2 |  | ST 41 | Jiexi | 1 |


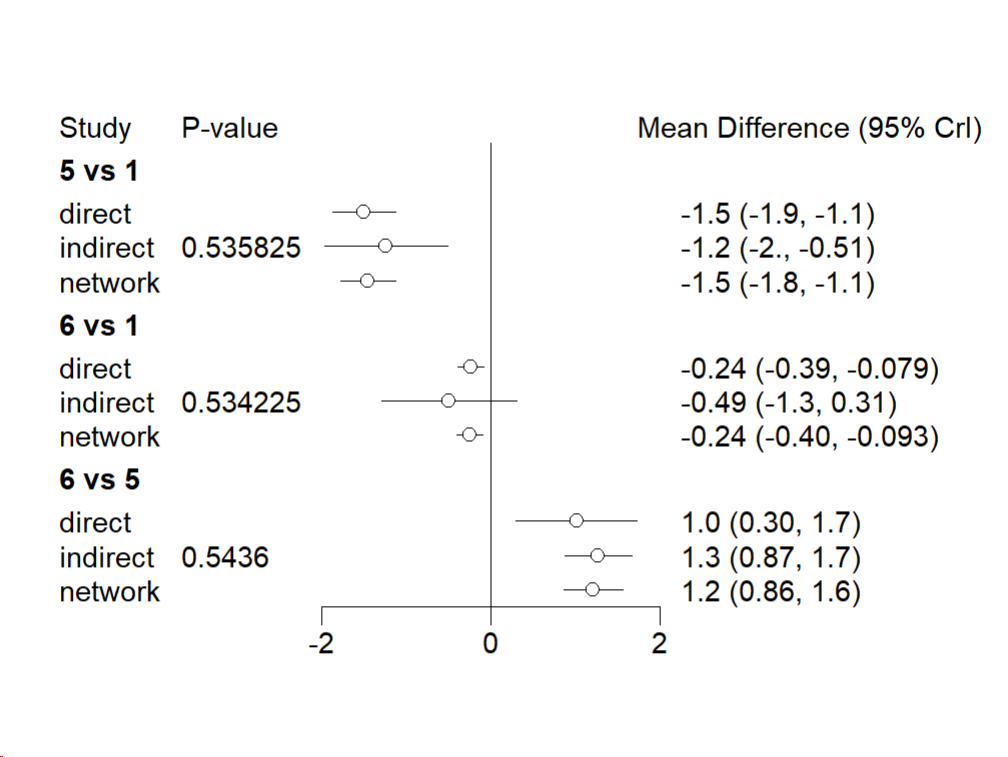

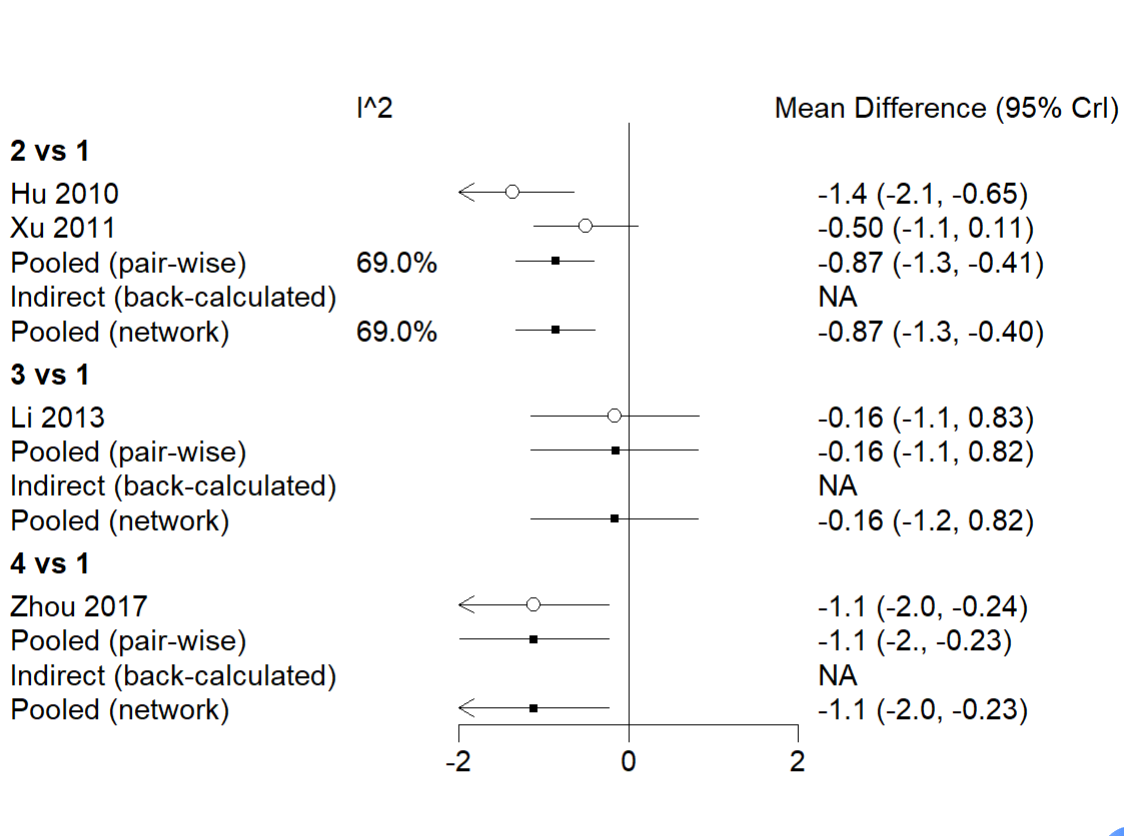

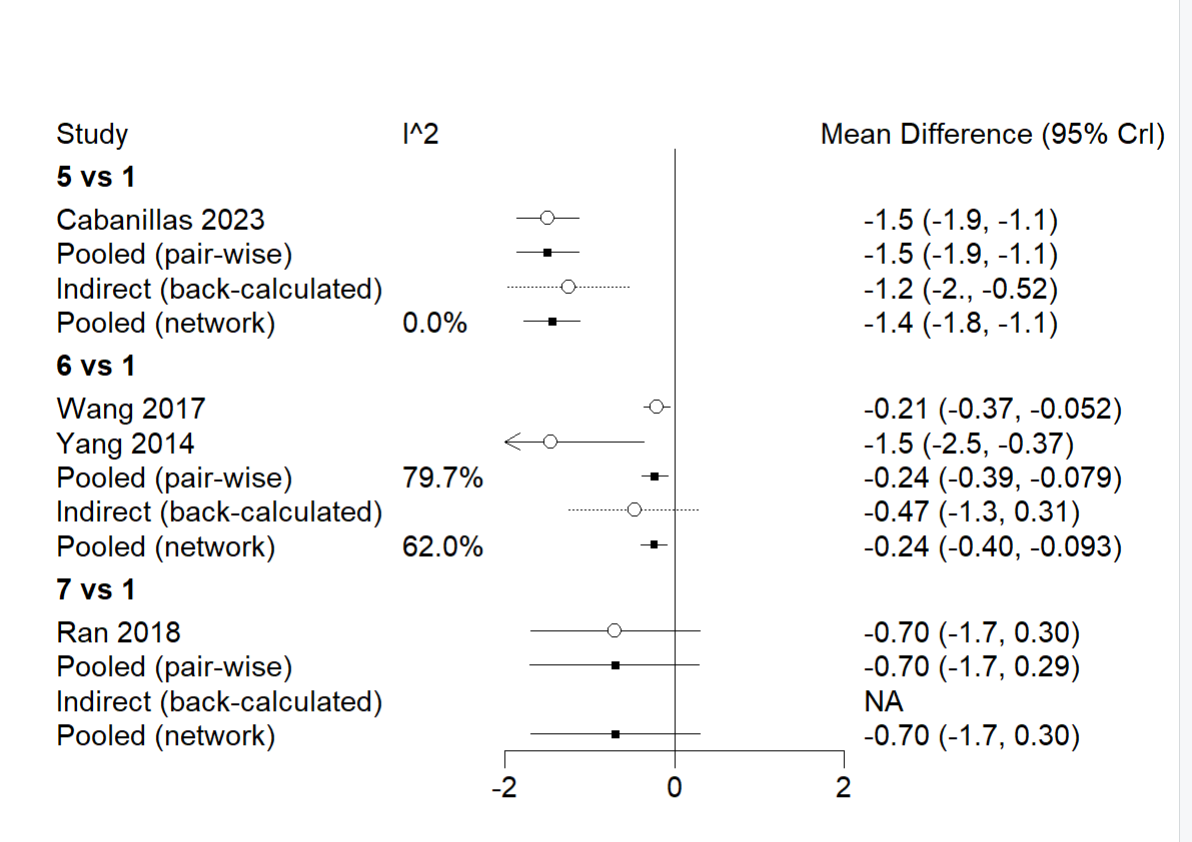


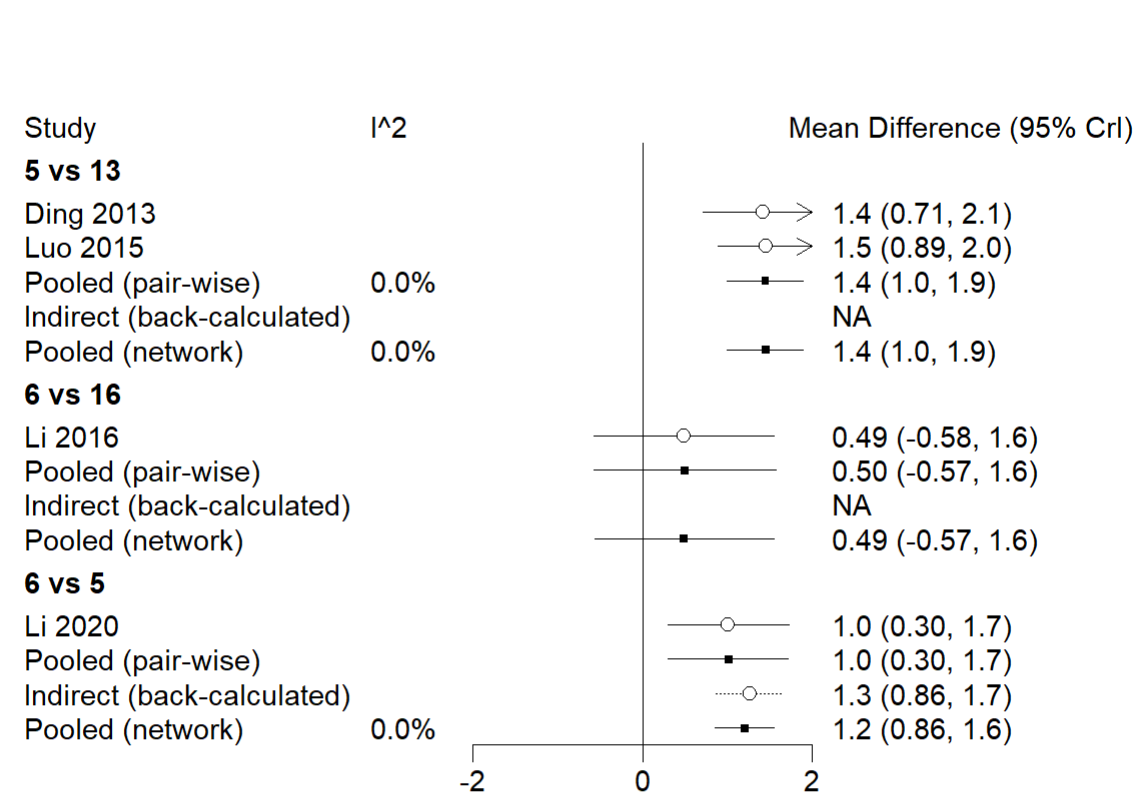


**Supplementary Figure 1** Inconsistency test and heterogeneity test of EDSS


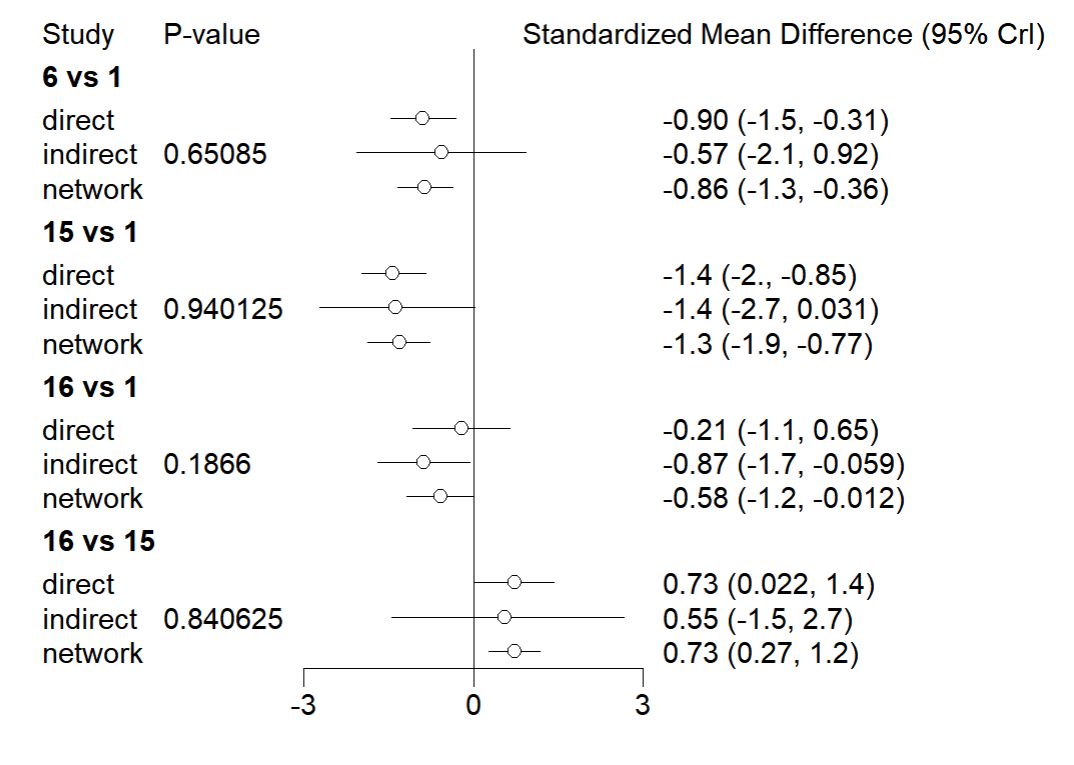

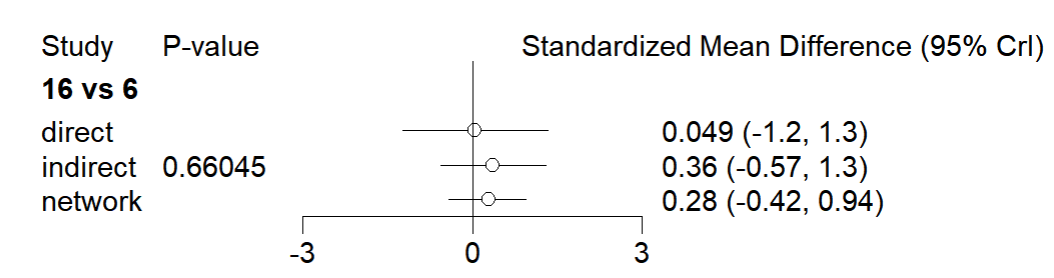

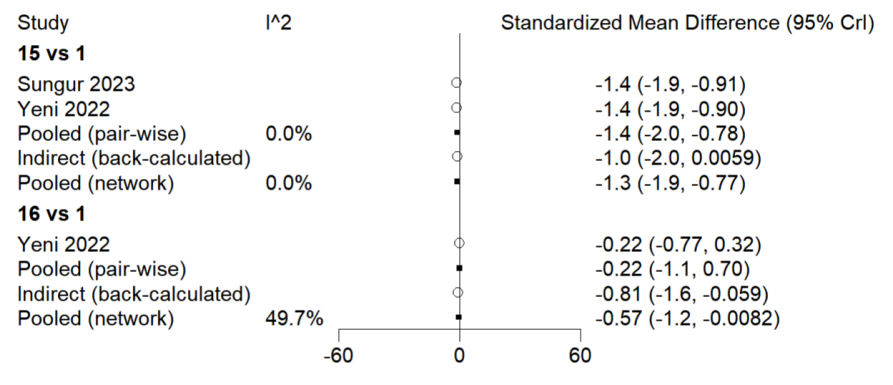

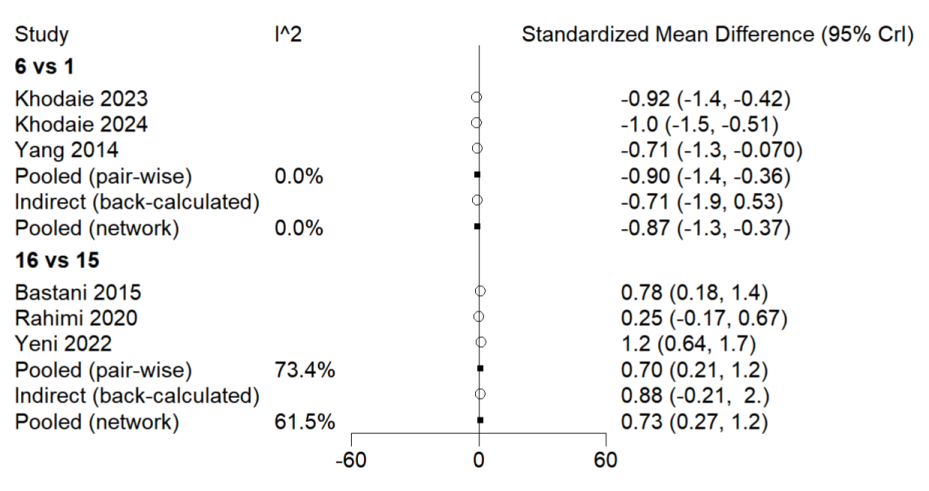

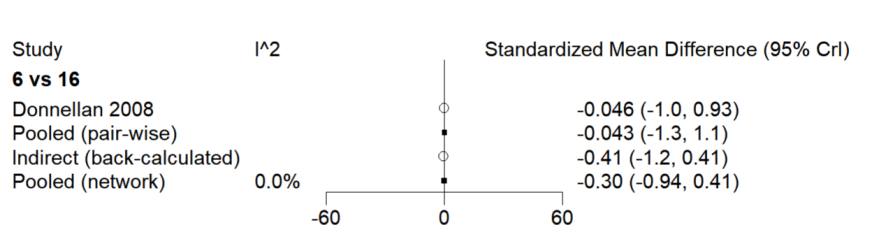


**Supplementary Figure 2** Inconsistency test and heterogeneity test of FSS


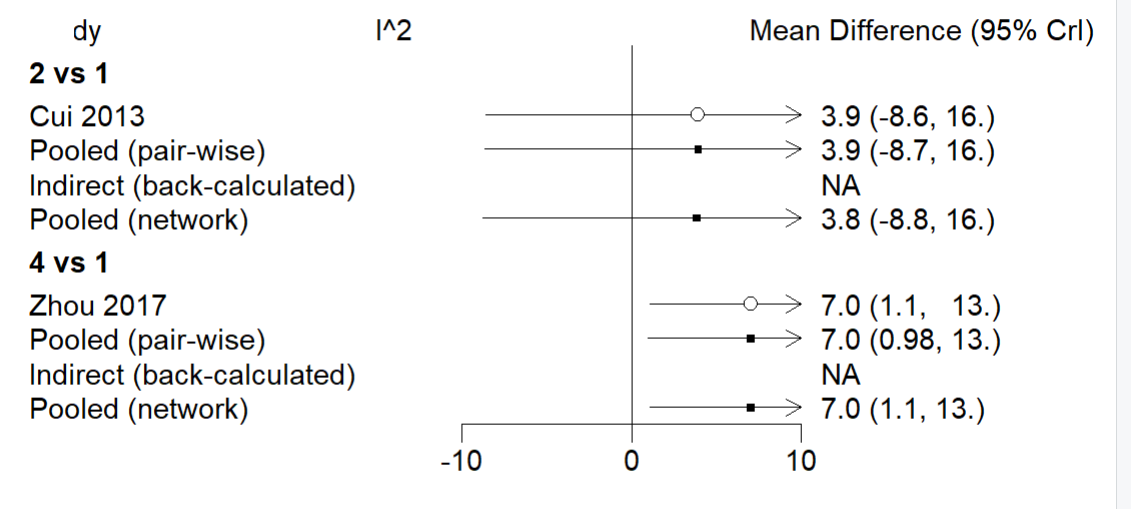


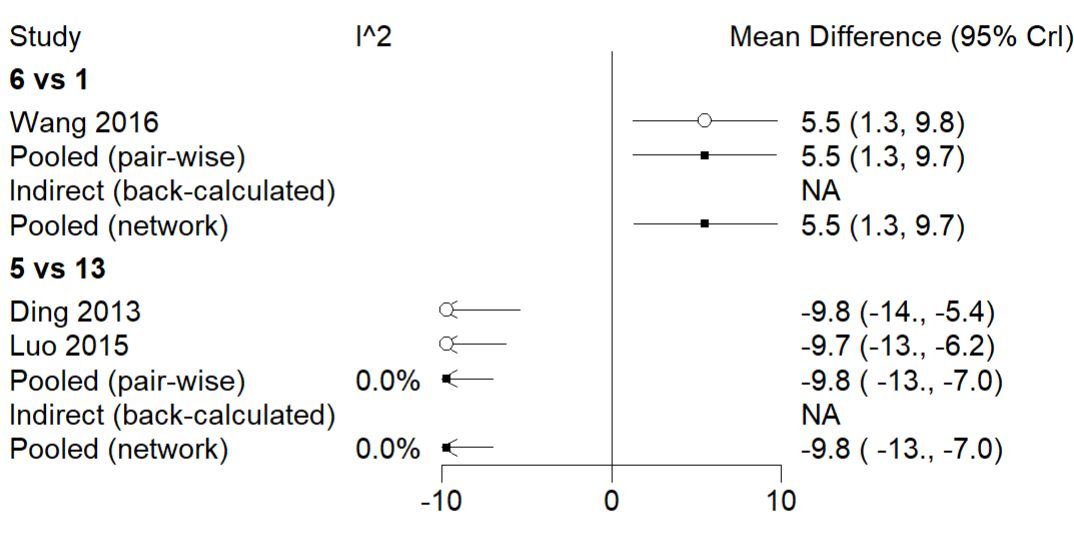

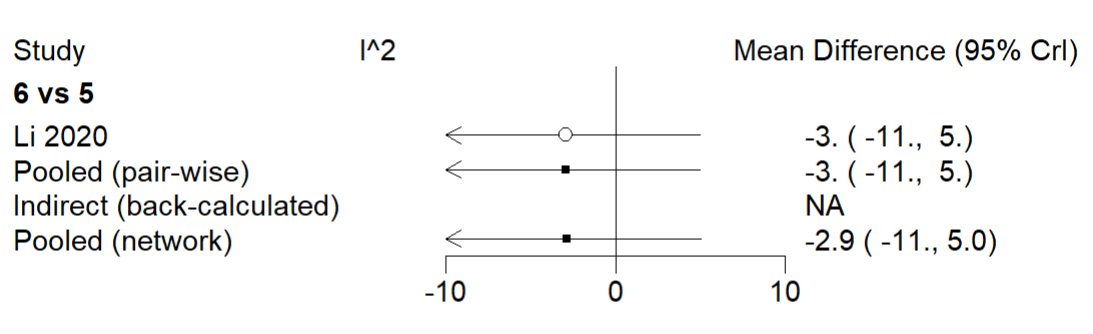


**Supplementary Figure 3** Heterogeneity test of Barthel index


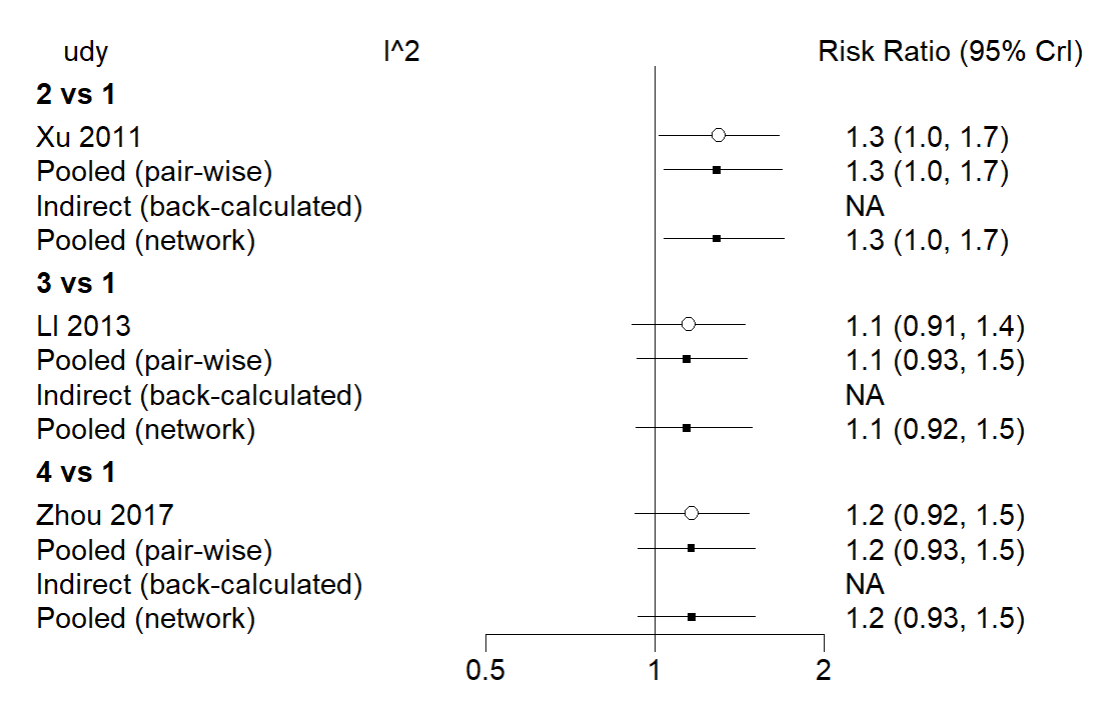

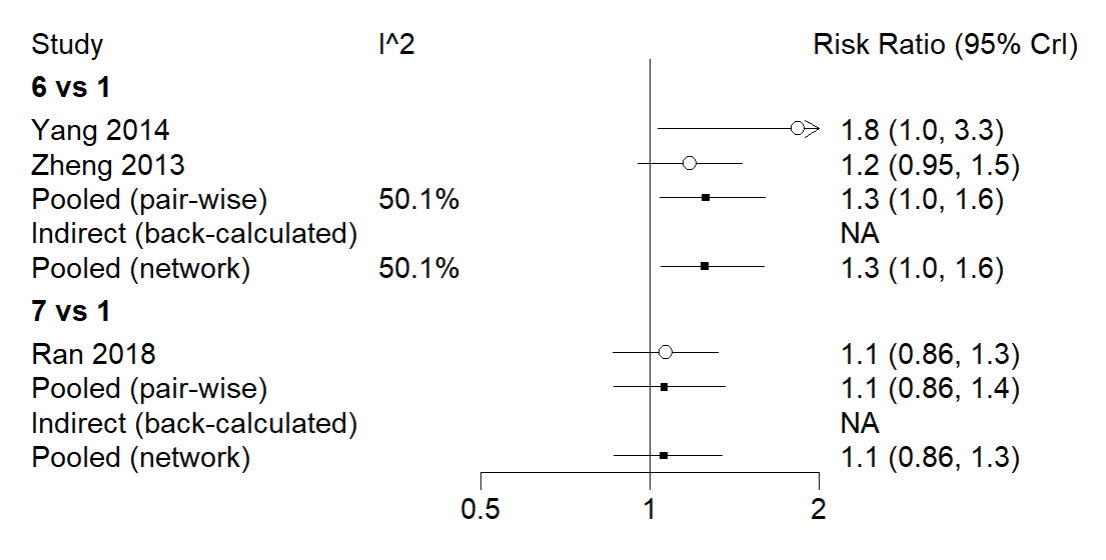

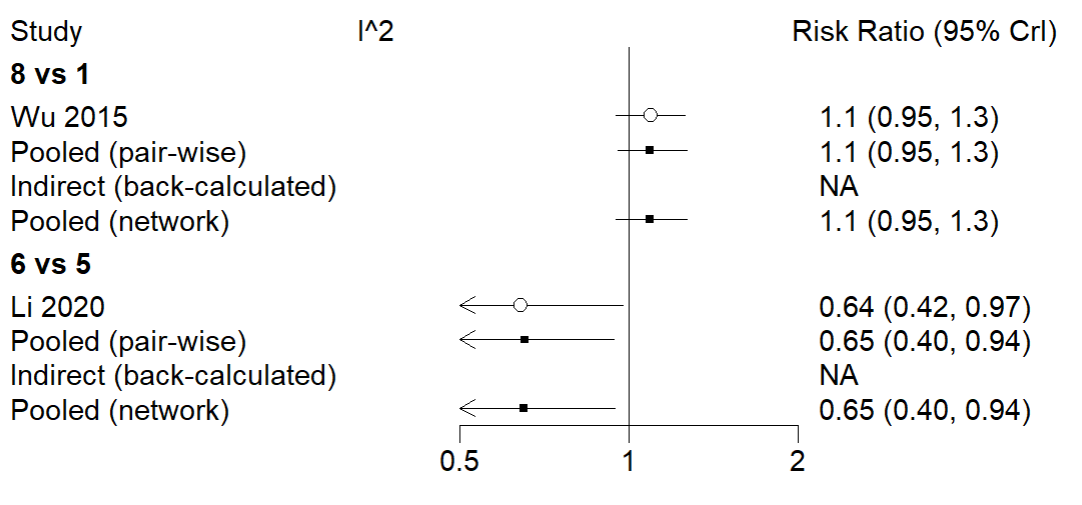


**Supplementary Figure 4** Heterogeneity test of treatment response rates
