# Supplementary material for: A network meta-analysis of the performance of acupoint stimulation therapy in improving fatigue, neurological function, and activities of daily living in patients with multiple sclerosis
Source: Front Neurol. 2026 May 26;17:1796876. doi: 10.3389/fneur.2026.1796876 (PMC13247309; doi:10.3389/fneur.2026.1796876)
Supplement: Supplementary file 2 [file Supplementary_file_2.docx]

**Supplementary Table 5** Risk of bias assessment for included studies

| Study | 1 | 2 | 3 | 4 | 5 | 6 | 7 | 8 | 9 | 10 | 11 | 12 | 13 | 14 | all |
| --- | --- | --- | --- | --- | --- | --- | --- | --- | --- | --- | --- | --- | --- | --- | --- |
| Xu 2011 | ✔️ | ❓ | ❓ | ❌ | ❓ | ✔️ | ✔️ | ✔️ | ❓ | ❌ | ✔️ | ❌ | ✔️ | ✔️ | poor |
| Hu 2010 | ✔️ | ❓ | ❓ | ❌ | ❌ | ✔️ | ✔️ | ✔️ | ❓ | ❓ | ✔️ | ❌ | ✔️ | ✔️ | poor |
| Cui 2013 | ✔️ | ❌ | ❓ | ❌ | ❓ | ✔️ | ✔️ | ✔️ | ❓ | ❓ | ✔️ | ❌ | ✔️ | ✔️ | poor |
| Wang 2017 | ✔️ | ✔️ | ❓ | ❌ | ✔️ | ✔️ | ✔️ | ✔️ | ❓ | ✔️ | ✔️ | ❌ | ✔️ | ✔️ | good |
| Li 2016 | ✔️ | ❓ | ❓ | ❌ | ❓ | ✔️ | ✔️ | ✔️ | ❓ | ✔️ | ✔️ | ❌ | ✔️ | ✔️ | Fair |
| Li 2013 | ✔️ | ❌ | ❌ | ❌ | ❓ | ✔️ | ✔️ | ✔️ | ❓ | ✔️ | ✔️ | ❌ | ✔️ | ✔️ | poor |
| Zhou 2017 | ✔️ | ❓ | ❓ | ❌ | ❓ | ✔️ | ✔️ | ✔️ | ❓ | ✔️ | ✔️ | ❌ | ✔️ | ✔️ | Fair |
| Ding 2013 | ✔️ | ✔️ | ❓ | ❌ | ❓ | ✔️ | ✔️ | ✔️ | ❓ | ✔️ | ✔️ | ❌ | ✔️ | ✔️ | Fair |
| Li 2020 | ✔️ | ❓ | ❌ | ❌ | ❓ | ✔️ | ✔️ | ✔️ | ❓ | ✔️ | ✔️ | ❌ | ✔️ | ✔️ | Fair |
| Yang 2014 | ✔️ | ❌ | ❌ | ❌ | ❓ | ✔️ | ✔️ | ✔️ | ❓ | ❌ | ✔️ | ❌ | ✔️ | ✔️ | poor |
| Zheng 2013 | ✔️ | ❓ | ❓ | ❌ | ❓ | ❓ | ✔️ | ✔️ | ❓ | ❓ | ✔️ | ❌ | ✔️ | ✔️ | poor |
| Luo 2015 | ✔️ | ❓ | ❓ | ❌ | ❓ | ✔️ | ✔️ | ✔️ | ❓ | ✔️ | ✔️ | ❌ | ✔️ | ✔️ | Fair |
| Wang 2016 | ✔️ | ✔️ | ❓ | ❌ | ❓ | ✔️ | ✔️ | ✔️ | ❓ | ✔️ | ✔️ | ❌ | ✔️ | ✔️ | Fair |
| Wu 2015 | ✔️ | ❓ | ❓ | ❌ | ❓ | ❓ | ✔️ | ✔️ | ❓ | ❓ | ❌ | ❌ | ✔️ | ✔️ | poor |
| Ran 2018 | ✔️ | ❓ | ❓ | ❌ | ❓ | ✔️ | ✔️ | ✔️ | ❓ | ❓ | ✔️ | ❌ | ✔️ | ✔️ | Fair |
| Cabanillas 2012 | ✔️ | ✔️ | ❓ | ✔️ | ✔️ | ✔️ | ✔️ | ✔️ | ✔️ | ✔️ | ✔️ | ❌ | ✔️ | ✔️ | good |
| Yeni 2022 | ✔️ | ✔️ | ❓ | ✔️ | ❓ | ✔️ | ✔️ | ✔️ | ✔️ | ✔️ | ✔️ | ✔️ | ✔️ | ❓ | good |
| Rahimi 2020 | ✔️ | ✔️ | ✔️ | ✔️ | ✔️ | ✔️ | ✔️ | ✔️ | ❓ | ✔️ | ✔️ | ✔️ | ✔️ | ❌ | good |
| Khodaie 2024 | ✔️ | ✔️ | ✔️ | ✔️ | ✔️ | ✔️ | ✔️ | ✔️ | ✔️ | ✔️ | ✔️ | ✔️ | ✔️ | ✔️ | good |
| Khodaie 2023 | ✔️ | ✔️ | ✔️ | ❌ | ✔️ | ✔️ | ✔️ | ✔️ | ✔️ | ✔️ | ✔️ | ✔️ | ✔️ | ✔️ | good |
| Bastani 2015 | ✔️ | ✔️ | ❓ | ✔️ | ✔️ | ✔️ | ✔️ | ✔️ | ✔️ | ✔️ | ✔️ | ✔️ | ✔️ | ✔️ | good |
| Donnellan 2008 | ✔️ | ✔️ | ✔️ | ✔️ | ✔️ | ✔️ | ✔️ | ❌ | ✔️ | ✔️ | ✔️ | ❌ | ✔️ | ✔️ | Fair |
| Sungur 2023 | ✔️ | ❌ | ❓ | ❓ | ❌ | ✔️ | ✔️ | ✔️ | ✔️ | ✔️ | ✔️ | ✔️ | ✔️ | ✔️ | Fair |

1. Was the study described as randomized, a randomized trial, a randomized clinical trial, or an RCT?

2. Was the method of randomization adequate (i.e., use of randomly generated assignment)?

3. Was the treatment allocation concealed (so that assignments could not be predicted)?

4. Were study participants and providers blinded to treatment group assignment?

5. Were the people assessing the outcomes blinded to the participants' group assignments?

6. Were the groups similar at baseline on important characteristics that could affect outcomes (e.g., demographics, risk factors, co-morbid conditions)?

7. Was the overall drop-out rate from the study at endpoint 20% or lower of the number allocated to treatment?

8. Was the differential drop-out rate (between treatment groups) at endpoint 15 percentage points or lower?

9. Was there high adherence to the intervention protocols for each treatment group?

10. Were other interventions avoided or similar in the groups (e.g., similar background treatments)?

11. Were outcomes assessed using valid and reliable measures, implemented consistently across all study participants?

12. Did the authors report that the sample size was sufficiently large to be able to detect a difference in the main outcome between groups with at least 80% power?

13. Were outcomes reported or subgroups analyzed prespecified (i.e., identified before analyses were conducted)?

14. Were all randomized participants analyzed in the group to which they were originally assigned, i.e., did they use an intention-to-treat analysis?
